# Viral microRNAs Target a Gene Network, Inhibit STAT Activation, and Suppress Interferon Responses

**DOI:** 10.1038/srep40813

**Published:** 2017-01-19

**Authors:** Dhivya Ramalingam, Joseph M. Ziegelbauer

**Affiliations:** 1 HIV and AIDS Malignancy Branch, National Cancer Institute, National Institutes of Health, Bethesda, Maryland 20892, USA

## Abstract

Kaposi’s sarcoma-associated herpesvirus (KSHV) encodes 12 pre-microRNAs during latency that are processed to yield ~25 mature microRNAs (miRNAs). We were interested in identifying cellular networks that were targeted by KSHV-miRNAs and employed network building strategies using validated KSHV miRNA targets. Here, we report the identification of a gene network centering on the transcription factor- signal transducer and activator of transcription 3 (STAT3) that is targeted by KSHV miRNAs. KSHV miRNAs suppressed STAT3 and STAT5 activation and inhibited STAT3-dependent reporter activation upon IL6-treatment. KSHV miRNAs also repressed the induction of antiviral interferon-stimulated genes upon IFNα- treatment. Finally, we observed increased lytic reactivation of KSHV from latently infected cells upon STAT3 repression with siRNAs or a small molecule inhibitor. Our data suggest that treatment of infected cells with a STAT3 inhibitor and a viral replication inhibitor, ganciclovir, represents a possible strategy to eliminate latently infected cells without increasing virion production. Together, we show that KSHV miRNAs suppress a network of targets associated with STAT3, deregulate cytokine-mediated gene activation, suppress an interferon response, and influence the transition into the lytic phase of viral replication.

Kaposi’s sarcoma-associated herpesvirus (KSHV) is a γ-herpesvirus (HHV-8) that is associated with Kaposi’s sarcoma (KS) and two lymphoproliferative disorders- primary effusion lymphoma (PEL) and multicentric Castleman’s disease (MCD). Infection with KSHV, like all herpesviral infections, progresses through a latent and a lytic phase. During latency, KSHV persists in the host with restricted gene expression and evades immune recognition. In contrast, the lytic phase of KSHV infection involves expression of all viral genes, infectious virion production and infected cell death. The KSHV latent genes confer anti-apoptotic, inflammatory and angiogenic benefits to the infected cell. The major latent genes expressed by KSHV are the latency-associated nuclear antigen (LANA), v-cyclin, v-FLIP, the Kaposin proteins and 12 pre-microRNAs^1^,^2^,^3^.

MicroRNAs (miRNAs) are small ~22-nt long RNAs that regulate gene expression post-transcriptionally. Typically, the “seed region” or nucleotides 2-8 from the 5′ end of miRNAs binds with imperfect complementarity to the 3′-untranslated regions (3′UTR) of their target mRNAs and mediates mRNA degradation or translational inhibition^4^. However, miRNAs can also repress mRNAs via interactions that depart from the canonical seed-matching interactions^5^,^6^. KSHV encoded pre-miRNAs are processed in the same manner as cellular miRNAs and yield at least 25 mature miRNAs whose functional significance in the context of KSHV infection are emerging. The percent of KSHV miRNAs over all small RNAs in infected PEL cell lines can range from ~25% (BC-1 and BCBL-1) to nearly 67% in BC-3 cells, and hence can impact a wide range of cellular processes in the context of infection^7^. Indeed, KSHV encoded miRNAs have been demonstrated to inhibit apoptosis, evade host immune responses or induce lytic reactivation (for reviews, refer to 8, 9, 10). Some targets of these KSHV-encoded miRNAs have been identified using bioinformatics-based identification of miRNA binding sites in the 3′UTRs of mRNAs^11^,^12^, microarrays^13^,^14^, proteomics^15^ and deep-sequencing techniques like cross-linking and immunoprecipitation^7^,^16^,^17^. We were interested in identifying specific cellular networks that are targeted by KSHV miRNAs. Using gene network interaction analysis of recently validated miRNA targets, we identified a signaling network with the transcription factor, STAT3 (signal transducer and activator of transcription 3), as a miRNA target that is also regulated by other KSHV miRNA targets.

STAT3 is a latent transcription factor that is activated in response to cytokine-induced stimuli. Cytokine engagement to their receptors activates the receptor-associated Janus kinases (JAK) that phosphorylate STAT3 on a critical tyrosine residue (Y705). The Y705-phosphorylated STAT3 (pY705-STAT3) molecules dimerize, translocate into the nucleus where they bind to promoter regions of their target genes and activate their transcription. STAT3 also gets phosphorylated on its C-terminal serine residue (pS727-STAT3); this process can be mediated by many kinases like PKCδ^18^ and IRAK1^19^. STAT3 is also a key player in the innate immune response involving type-I interferons (IFN) like IFN-α/β to counteract viral infections. Binding of IFNs to their receptors leads to the phosphorylation of various STAT proteins including STAT1 and STAT3. This is followed by the formation of STAT complexes that can promote the expression of inflammatory cytokines and antiviral interferon-stimulated genes (ISGs)^20^.

This network of KSHV miRNA targets centering on STAT3 also contained several novel KSHV miRNA targets like erythropoietin receptor (EPOR), hepatocyte growth factor receptor (MET), the antiapoptotic protein-baculoviral IAP repeat containing 5 (BIRC5), growth arrest and DNA damage 45 family member (GADD45β) and protein kinase C-δ (PRKCD or PKCδ). STAT3 was one of the most down-regulated proteins in proteomic screens performed to identify KSHV miRNA targets^15^. Other validated KSHV miRNA targets such as interleukin-1 receptor- associated kinase 1 (IRAK1) and GRB2 were also present in this network^15^,^21^. Further, we observed that HUVECs that were transfected with KSHV miRNAs that repress STAT3 demonstrated a weakened response to cytokines such as IL6 and the antiviral interferon, IFN-type A (also known as IFN-αA or IFN-α2 A) STAT3 repression using siRNAs or with chemical inhibitors in latently infected BCBL-1 cells promoted entry into the lytic phase of the viral life cycle. Together, our results show that KSHV miRNAs suppress gene expression in this STAT3 network, inhibit cytokine-mediated gene activation, defend against antiviral interferon responses, and regulate lytic reactivation.

## Results

### miRNA-target analysis reveals canonical and non-canonical base-pairing interactions in the 3′UTRs

**Figure 1:**
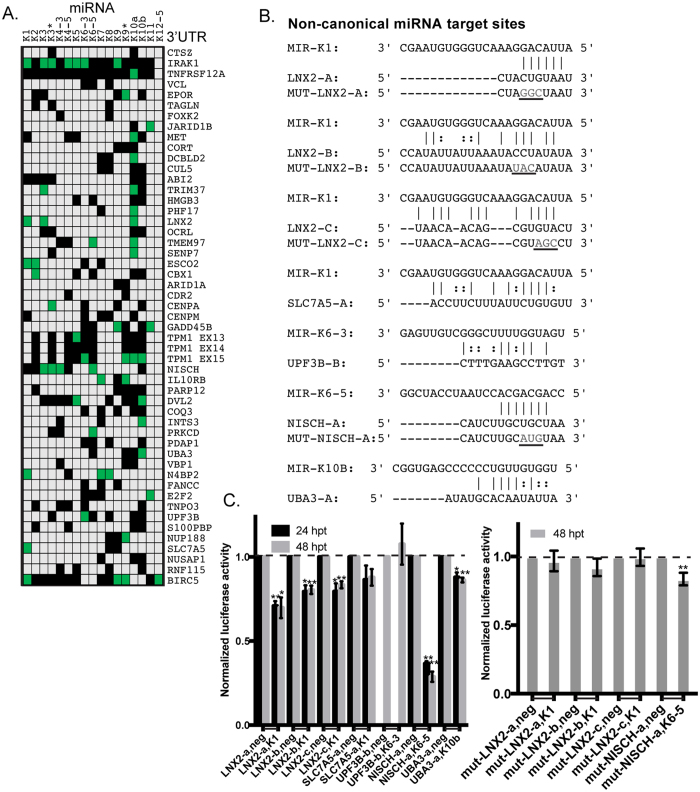
3′UTR luciferase assays for predicted targets of KSHV-encoded miRNAs. (**A**) 293 cells were transfected with 3′UTR luciferase reporter plasmids for the indicated genes along with KSHV miRNA mimics. Cells were harvested at 48 hpt and reporter activity was measured as described in Methods. The table highlights conditions where the luciferase reporter was repressed (p < 0.05) in green boxes. Gray boxes denote untested conditions and black boxes represent tested conditions where no significant repression was observed. (**B**) Potential non-canonical miRNA targets were identified using miRanda^25^ and PAR-CLIP sequences^7^. Mutated bases (“MUT”) are denoted in gray text and underlined. (**C**) Luciferase assays with the sites in (**B**) are shown. Activity with each miRNA was normalized to a negative control miRNA (Neg) with each reporter (denoted by joining horizontal line on x-axis). Error bars show standard deviation from three biological replicates. *Represents p < 0.05 and ** is p < 0.01.

Using a combination of microarray and proteomic approaches with gain and loss of viral miRNA activity, we have previously reported the identification of numerous mRNA targets of KSHV-encoded miRNAs (BCLAF1, TNFRSF12A, IRAK1, STAT3, TPM1)^13^,^15^,^21^,^22^. Using these previous expression datasets, we integrated the data using a rank sum method as described previously^23^ in order to select genes with the highest expression changes in response to gain or loss of viral miRNA function. Importantly, in this analysis, we ignored miRNA seed-matching information and ranked potential miRNA targets solely by their expression changes in response to individual KSHV miRNAs and KSHV infection. We then cloned 49 different full-length 3′UTRs of these predicted miRNA target mRNAs (genes changing the most in the microarray analysis described above) downstream of a *renilla luciferase* gene into a reporter plasmid (pDEST-765) and performed reporter assays in cells in the presence of various KSHV miRNAs. Luciferase activity was normalized to control assays that used parental luciferase reporters (lacking inserted human 3′UTR sequences) and non-targeting negative control miRNA mimics. These data demonstrated that 28 of 49 3′UTRs were repressed by at least one viral miRNA, when using a t-test and a p-value cutoff of 0.05 (Fig. 1A, Complete data in Table S1). We did not use an arbitrary fold-change cut-off value to denote repression, but used a standard t-test to assess the mean fold-change and standard deviation within the same test. However, this screening is likely to be an underestimate of miRNA targets, since we did not test all combinations of all individual viral miRNAs and 3′UTRs. There were also several instances of a single 3′UTR being repressed by multiple KSHV miRNAs (e.g. IRAK1, TPM1), indicating a high level of redundancy in KSHV miRNAs mediated targeting of cellular mRNAs. Importantly, these data also validate dozens of new miRNA targets of viral miRNAs.

There were at least 50 instances of a miRNA repressing a 3′UTR (green boxes, Fig. 1A), but the majority (28 of 50) of these target interactions were missed using a common bioinformatic tool for predicting canonical miRNA targets^24^. Here we use the definition of canonical miRNA sites to include “7 mer-m8”, “7 mer-A1”, and “8 mer” sites. These results suggested that some miRNA:target repression events may function through non-canonical sites, as has been demonstrated for human miRNAs^6^. Using the miRNA target prediction program miRanda^25^ with customized parameters, we were able to predict the existence of non-canonical miRNA binding sites in the 3′UTRs of many mRNAs. Indeed, integration with PAR-CLIP data^7^ identified Ago2-associated sequences within the 3′UTRs of mRNAs that do not contain perfect seed-matching sites to these miRNAs. These binding sites had 5 mer/6 mer miRNA binding regions (e.g. LNX2-a), deviations from the 2–7 seed-matching region (e.g. NISCH-a), and several G:U interactions spread across the length of the miRNA (e.g. LNX-b, UBA3-a) (Fig. 1B). To validate these non-canonical miRNA binding sites, we introduced these potential sites into pDEST-765 and repeated the 3′UTR luciferase reporter assays in the presence of KSHV miRNAs. We were able to identify functional miRNA binding sites in the 3′UTRs of genes like LNX2, UBA3, and NISCH (Fig. 1C). We observed that the LNX2 3′UTR contained three potential non-canonical binding sites for KSHV-miR-K1 (Fig. 1B) and all three sites appeared to be involved in miRNA-mediated repression (Fig. 1C). Interestingly, luciferase activity of the non-canonical site identified in the NISCH 3′UTR, where the binding was mediated by nucleotides 3–9 of KSHV miR-K6-5, was repressed by nearly 75% compared to the control miRNA, suggesting strong interactions at this site. We further investigated the most repressed miRNA targets from Fig. 1C by mutating three bases in each target site. Results from mutating these miRNA sites indicated that the mutated reporters were no longer repressed by the appropriate miRNA for the three different sites from the LNX2 3′UTR. A similar mutation in the NISCH site did not render the mutant reporter resistant to repression, but the repression by miR-K6-5 was severely diminished (16% repression with mutant) when compared the 71% repression observed with wild-type reporter. This indicates that bases outside of the mutated region also are important for the repression of the miR-K6-5 site in the NISCH 3′UTR. These non-canonical miRNA binding sites demonstrate the existence of functional sites that can be missed by scanning only for 7 mer/8 mer interactions in the 3′UTRs. Furthermore, a single 3′UTR can be targeted at multiple sites by multiple KSHV miRNAs, using both canonical and non-canonical interactions, demonstrating a high level of complexity in their regulation. These validated targets may be of interest in future studies of the functional roles of viral miRNAs.

A single miRNA can repress gene expression from tens to hundreds of target mRNAs. Further, a single 3′UTR can have binding sites for multiple miRNAs^4^. We speculated that the KSHV miRNAs could thus repress multiple mRNAs across many biological pathways. We were interested in identifying specific networks of cellular genes that were particularly enriched for viral miRNA targets. After combining validated miRNA targets identified by microarrays (Fig. 1) and proteomics (STAT3)^15^, we performed a network interaction analysis using 34 validated KSHV miRNA targets using MetaCore^TM^ software. This analysis utilized manually-curated associations of genes obtained from peer-review literature. Specifically, we used the software to investigate only direct interactions (e.g. phosphorylation, transcription regulation) between miRNA target genes. These specific interactions and supporting references are detailed in Table S3.

### KSHV miRNAs repress multiple mRNAs in the STAT3 signaling network and suppress STAT3 activation upon IL6 treatment

**Figure 2:**
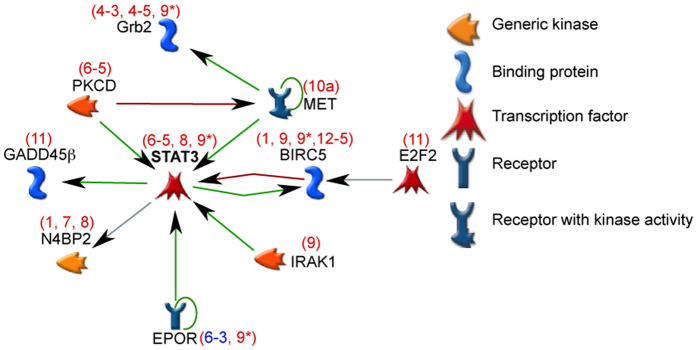
Multiple KSHV miRNA targets regulate STAT3 activity. Protein network analysis performed with validated miRNA targets validated in Fig. 1 using MetaCore reveals the existence of an interaction network centering on STAT3. Green arrows indicate positive regulation of STAT3 by the indicated protein while red arrows indicate inhibition. STAT3, itself a KSHV miRNA target, can be activated by indicated miRNA targets (e.g. IRAK1, MET). Several downstream targets of STAT3 can also be repressed by KSHV miRNAs. The name of the miRNA targeting the mRNA identified either in Fig. 1 (red) or from RT-qPCR assays (blue) are given on top of the target.

The largest network of directly associating proteins among the 34 miRNA targets consisted of 10 KSHV miRNA targets and centered on STAT3 (Fig. 2). All of the luciferase validations for these targets are included in Table S1. The interactions in this network are based on the publications shown in Table S3. Note this network of 10 genes is a subset of the genes described in Fig. 1 and did not contain the targets with non-canonical miRNA sites detailed in Fig. 1. The interaction network revealed that targets that stimulate STAT3 activity in normal cells- IRAK1, PKCδ, EPOR and MET, were also repressed by KSHV-encoded miRNAs (Fig. 1, Table S1). Some downstream targets of STAT3 such as BIRC5 and GADD45B were also repressed by KSHV miRNAs (Fig. 2). Since multiple activators of STAT3 activity and expression were identified, we hypothesized that KSHV miRNAs may regulate STAT3 activation.

**Figure 3:**
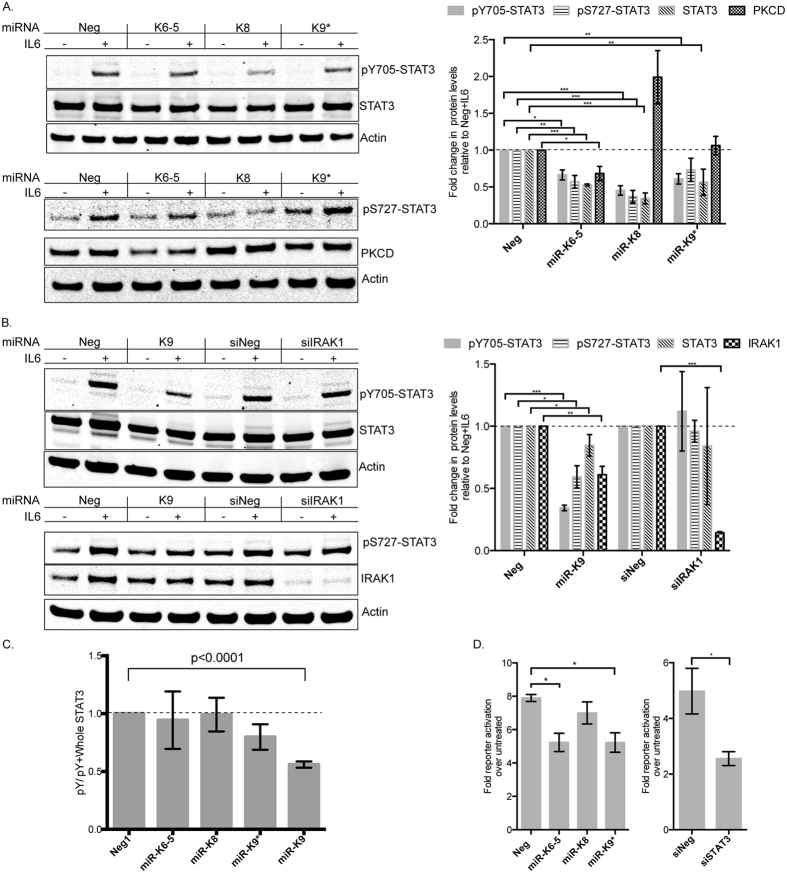
KSHV miRNAs inhibit IL6-dependent STAT3 activation and inhibit STAT3-dependent reporter activation. (**A**) HUVECs were transfected with KSHV miRNAs (miR-K6-5, miR-K8 or miR-K9*) or control miRNAs (Neg). At 48 hpt, transfected cells were mock-treated or treated with IL6 for 20 minutes. Equal amounts of protein were probed for pY705-STAT3 and total STAT3 (top) and pS727-STAT3 and PKCδ (bottom). Relative levels of various proteins were normalized to actin, compared to the control miRNA (Neg), and plotted on the right (n = 4). (**B**) HUVECs were transfected with miR-K9 (that targets IRAK1) or siIRAK1 along with appropriate negative control RNAs. Lysates were probed for pY705-STAT3, pS727-STAT3, total STAT3 and IRAK1. Relative levels of various proteins were normalized to actin and plotted on the right (n = 4). (**C**) The changes in the levels of pY705-STAT3 relative to total STAT3 were measured from 3 A and 3B and plotted (n = 3). (**D**) 293 cells were reverse-transfected with the 4xM67 pTATA-Luc, pRNL-TK control and KSHV miRNAs or siRNAs. Cells were left untreated or treated with IL6 for eight hours and relative luciferase levels were measured as described in the Methods (n = 3). Full-length blots are in Figure S2.

To study how the repression of these different targets by miRNAs affected STAT3 function, we transfected various KSHV miRNAs into HUVECs and measured STAT3 activation upon IL6-treatment. Previously, we have demonstrated, that STAT3 is repressed by KSHV miRs-K6-5, -K8 and –K9*^15^. HUVECs were transfected with miR-K6-5, -K8 and –K9*, following which they were treated with IL6 and levels of pY705-STAT3, pS727-STAT3 and unphosphorylated STAT3 were measured. In cells transfected with negative control miRNA, IL6 treatment resulted in ~10-fold increase in the levels of pY705-STAT3 over untreated controls (Fig. 3A). However, cells transfected with KSHV miRNAs, miR-K6-5, -K8 and –K9*, showed reduced levels of pY705-STAT3 upon IL6-treatment (Fig. 3A). Upon IL6-treatment, miR-K12-6-5 and miR-K12-8, also had a ~2-fold reduction in pS727-STAT3 levels (Fig. 3A, bottom panel). We note that we previously measured miRNAs associated with the RNA-induced silencing complex (RISC) as RISC-associated miRNAs are a more accurate measure of functional intracellular miRNA levels^26^. We have previously demonstrated that the transfected KSHV miRNA-mimics are associated with RISC at levels that are comparable to those after *de novo* KSHV infection^15^. Together, these results and previous STAT3 3′UTR results^15^ demonstrated that KSHV miRNAs could repress STAT3 activation upon IL6-treatment, likely through a combination of targeting STAT3 mRNA and also currently unknown upstream activators of STAT3.

### KSHV miRNAs repress Ser/Thr kinases that regulate STAT3

From the 3′UTR luciferase assays (Fig. 1), we observed that miR-K6-5 strongly repressed the 3′UTR of PKCδ, a Ser/Thr kinase can phosphorylate STAT3^18^. We measured the levels of PKCδ in HUVECs transfected with miR-K6-5 and observed strong repression in its protein levels (Fig. 3A). It is possible that PKCδ repression contributes the ~2-fold reduction in pS727-STAT3 levels observed upon IL6-treatment. However, the exact mechanism of miR-K6-5-mediated suppression of STAT3 phosphorylation is unknown and may involve a number of miR-K6-5 targets. We observed that miR-K8 expression resulted in ~2-fold increase in the protein levels of PKCδ (Fig. 3A; right panel). This is likely due to indirect effects of miR-K8 targeting an inhibitor of PKCδ. However, it is to be noted that in our microarray analysis, RNA levels of PKCδ were repressed by ~30% (average log_2_ value of −0.47) in the context of KSHV infection, when compared to uninfected controls^13^.

A second Ser/Thr kinase, IRAK1, is also known to phosphorylate and activate STAT3^19^. IRAK1 is also a target of KSHV miR-K9^21^. To study the effect of IRAK1 suppression on STAT3 activation, HUVECs were transfected with miR-K9 and levels of pY705-STAT3 were measured upon IL6-treatment. We observed ~70% reduction in the levels of pY705-STAT3 levels in the presence of miR-K9 when compared to miR-Neg (Fig. 3B). There was also ~40% repression in pS727-STAT3 levels upon IL6-treatment, which could be a result of IRAK1 repression (~40% repression with miR-K9). No repression of pY705-and pS727-STAT3 were seen with siRNAs against IRAK1, despite robust repression of IRAK1, suggesting that miR-K9 repressed STAT3 activation primarily in an IRAK1-independent mechanism. While repression of pY705-STAT3 levels as a result of KSHV miRNAs could also be an outcome of reduced STAT3 protein levels, we observed for some cases, like miR-K9, that the fraction of phosphorylated STAT3 relative to total STAT3 was reduced (Fig. 3C).

We also measured the effect of KSHV miRNAs on the transcriptional activity of STAT3. We utilized a STAT3-driven firefly luciferase reporter plasmid along with KSHV miRs-K6-5, -K8 or –K9* and transfected 293 cells. As additional controls, siRNAs against STAT3 were used. Transfected 293 cells were treated with 20 ng/ml IL6 and fold activation of the reporter was measured relative to untreated controls (Fig. 3D). In miR-Neg transfected cells, IL6-treatment resulted in an 8-fold increase in reporter activation, while it was reduced to a 5-fold increase with miR-K6-5 and miR-K9*. However, miR-K8 did not affect reporter activation. siNeg-transfected cells showed ~5-fold increase in reporter activity upon IL6-treatment, while in the siSTAT3-transfected cells, the activity was reduced to ~2-fold. Together, these results suggest that KSHV encoded miRNAs inhibit STAT3 activation upon IL6-treatment and inhibit its transcriptional activity.

### STAT3 repression by KSHV miRNAs contributes to inhibition of interferon-mediated antiviral responses

**Figure 4:**
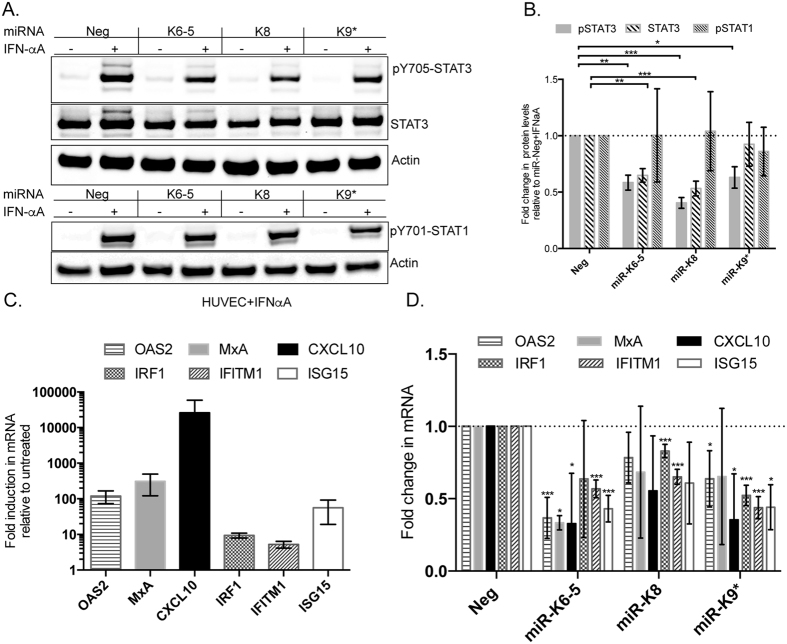
KSHV miRNAs inhibit IFN-αA-dependent STAT3 activation. (**A**) HUVECs were transfected with KSHV miRNAs (miR-K6-5, miR-K8 or miR-K9*) or control miRNAs (miR-Neg). At 48 hpt, transfected cells were mock-treated or treated with IFN-αA for 20 minutes. Equal amounts of protein were probed for pY705-STAT3, total STAT3 and actin (top) or pY701-STAT1 and actin (bottom). (**B**) Relative levels of various proteins from 4A were normalized to actin, compared to the negative control miRNA (Neg), and plotted. (**C**) HUVECs were transfected with miR-Neg and treated with IFN-αA for 4 hours and mRNA levels of the indicated interferon-stimulated genes (ISGs) were measured by qRT-PCR. D. HUVECs were transfected with miRNAs as in (**A**) and treated with IFN-αA for 4 h and qRT-PCR was performed to measure levels of the indicated ISGs. Expression changes in ISGs were normalized to miR-Neg+ IFN-αA condition and plotted. Statistical significance relative to corresponding control RNAs with at least three biological replicates and p < 0.05 is denoted by *p < 0.001 by and **p < 0.0001 by. ***Full-length blots are in Figure S3.

IFN-α can activate STAT1 as well as STAT3 to elicit antiviral responses^27^. We were interested in determining if KSHV miRNAs can suppress IFN-αA-mediated STAT3 activation and therefore, inhibit expression of ISGs. HUVECs were transfected with KSHV miRNAs following which they were treated with IFN-αA for 20 minutes. We measured changes in the levels of pY705-STAT3 and total STAT3 upon IFN-αA treatment. As an additional control, we also measured changes in the levels of pY701-STAT1 upon IFN-αA-treatment. In HUVECs transfected with miR-Neg control, IFN-αA-treatment resulted in ~20-fold activation in the levels of pY705-STAT3, compared to untreated controls (Fig. 4A, lanes 1 and 2). However, KSHV miRNA-transfected cells showed ~2-fold reductions in the levels of pY705-STAT3, when compared to miR-Neg controls (Fig. 4A), suggesting a strong inhibition of this pathway. IFN-αA-treatment also strongly activated STAT1 in HUVECs, however, KSHV miRNAs had less of an effect on overall pSTAT1 levels compared to pY705-STAT3, suggesting specificity in repression of STAT proteins (Fig. 4A, Figure S1).

IFN-αA has potent antiviral activity and can activate chemokines (e.g. CXCL10) and antiviral proteins (e.g. OAS2, MxA, ISG15, IFITM1, IRF1)^28^. To determine if KSHV miRNAs can suppress activation of IFN-αA –regulated genes via STAT3 repression, we transfected HUVECs with KSHV miRNAs and treated them for 4 hours with IFN-αA and measured changes in the levels of antiviral genes using qRT-PCR. HUVECs transfected with control miRNA, miR-Neg, demonstrated strong increases in the mRNA levels of the aforementioned ISGs upon IFN-αA treatment (Fig. 4C). However, upon IFN-αA treatment in the presence of KSHV miRNAs, we observed strong decreases in the activation of several ISGs. CXCL10, a ~10 kDa chemokine, was found to be consistently downregulated (~2-fold) by miR-K6-5 and –K9* (Fig. 4D). A ~2-fold decrease in activation of ISG15, IFITM1 and IRF1 mRNAs were also observed with other KSHV miRNAs that repress STAT3. KSHV miR-K6-5 also repressed the induction of antiviral ISGs- OAS2 and MxA. From Western blot analysis (Fig. 4A and B), it appeared that the KSHV miRNAs that repressed STAT3 did not affect STAT1 levels upon IFN-treatment. We conclude that KSHV miRNAs can inhibit ISG activation in the presence of interferons.

### KSHV miRNAs repress EPOR mRNA to inhibit EPO-dependent STAT5 activation

**Figure 5:**
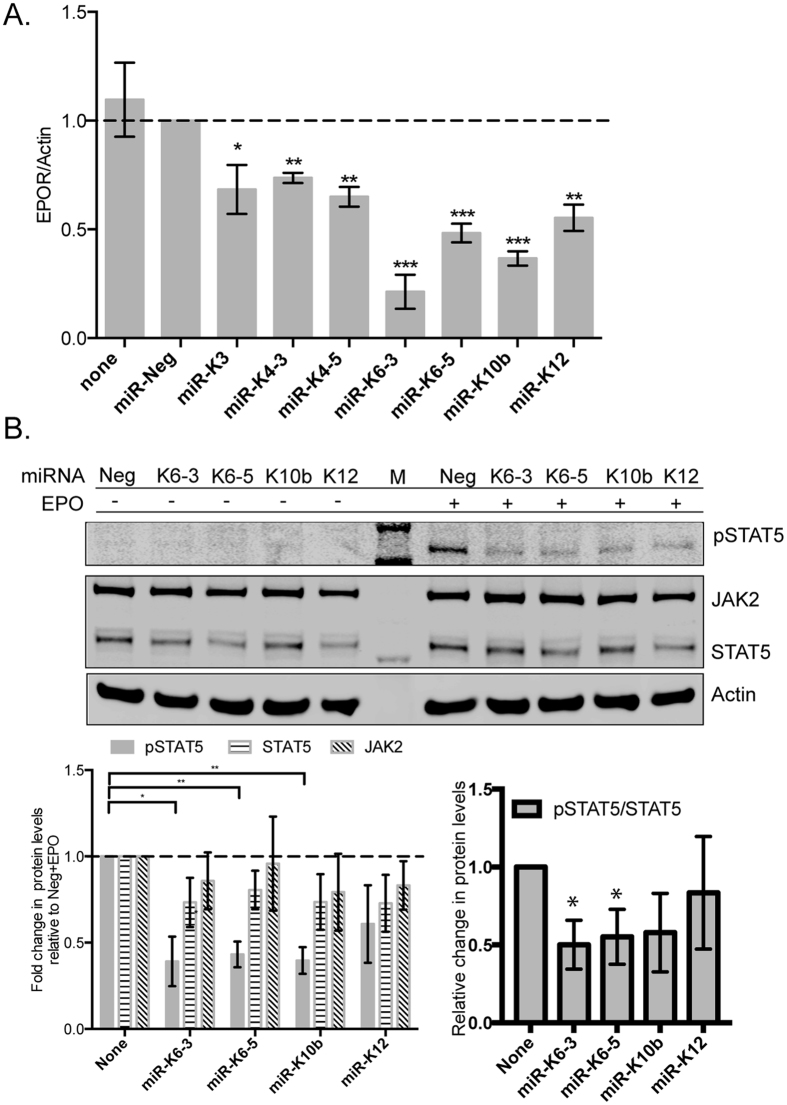
KSHV miRNAs repress the levels of erythropoietin-receptor (EPOR) mRNA and reduce erythropoietin-dependent STAT5 activation in endothelial cells. (**A**) HUVECs were transfected with individual KSHV miRNA mimics along with control miRNAs and the levels of EPOR mRNA were measured 48 hpt by qRT-PCR. B. HUVECs were transfected with KSHV miRNAs (miR-K6-3, miR-K6-5, miR-K10b or miR-K12) or miR-Neg. At 48 hpt, transfected cells were mock-treated or treated with EPO for 10 minutes. Equal amounts of protein were probed for pY694-STAT5, JAK2 and total STAT5. “M” denotes the protein size marker loaded in the center lane. Relative levels of various proteins were normalized to actin and quantified below. Full-length blots are in Figure S3.

From HUVEC mRNA panels, we identified KSHV miRNAs- K6-3, -K6-5 and -K10b as miRNAs that could strongly repress the levels of the EPOR mRNA (Fig. 5A). Binding of erythropoietin (EPO) to EPOR predominantly activates STAT5 as pY694-STAT5 (pSTAT5), but can also activate STAT3^29^. However, we were unable to demonstrate STAT3 activation upon EPO-treatment in endothelial cells (Figure S1). To test if KSHV miRNAs can inhibit STAT5 activation, we transfected KSHV miRNAs into HUVECs and treated these cells with EPO. STAT5 activation (as pSTAT5) was observed upon EPO-treatment in HUVECs transfected with miR-Neg (Fig. 5B). HUVECs transfected with KSHV miRs- K6-3, -K6-5 and –K10b showed ~2-fold reduction in the levels of pSTAT5 compared to miR-Neg-transfected cells, upon EPO-treatment. However, the levels of unphosphorylated STAT5 protein and JAK2 in these cells did not change dramatically in the presence of these miRNAs. Assessing the ratio of pSTAT5 to total STAT5 demonstrated that the majority of the changes were to phosphorylation of STAT5.

STAT3 and STAT5 are both activated in response to type-I interferons like IFN-α and therefore can regulate antiviral responses^20^. It is interesting that KSHV inhibits STAT5 activation in cell types like HUVECs, where STAT3 activation was not observed with EPO. Together, our results suggest a mechanism by which KSHV utilizes miRNAs to inhibit cytokine responses either directly by repression of STAT proteins (e.g. STAT3) or indirectly by repression of their cognate receptors (e.g. EPOR).

### BIRC5 repression by KSHV miRNAs promotes KSHV infection

**Figure 6:**
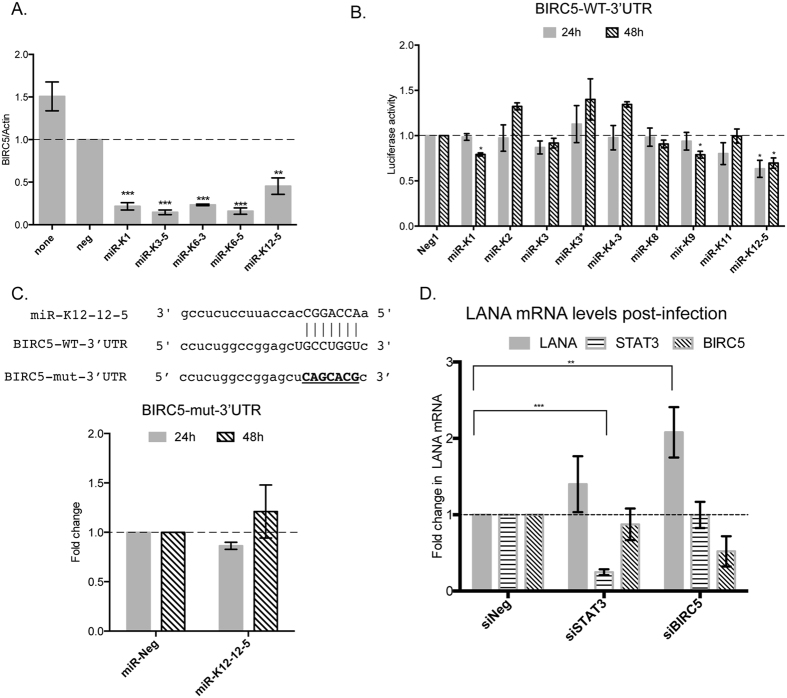
KSHV miRNAs directly repress the levels of the STAT3-regulated protein, BIRC5. (**A**) HUVECs were transfected with individual KSHV miRNA mimics along with control miRNAs and the levels of BIRC5 were measured 48 hpt by qRT-PCR. (**B**) 3′UTR luciferase reporter assays were performed using pDEST-BIRC5-3′UTR (BIRC5-WT-3′UTR). 293 cells were reverse-transfected with wild type (WT) pDEST-BIRC5-3′UTR and individual KSHV miRNA mimics. Relative luciferase expression was measured at 24 hpt and 48 hpt as described in the Methods. (**C**) Binding site for KSHV miR-K12-5 as predicted by the target site prediction software, miRanda is shown (top). This site was mutated using site-directed mutagenesis (bottom) to generate mutant, pDEST-BIRC5-mut-3′UTR. 3′UTR reporter assays were repeated in 293 cells using pDEST-BIRC5—mut-3′UTR with miR-K12-5 and luciferase levels were analyzed at 24 hpt and 48 hpt. D. HUVECs were transfected with siRNAs targeting STAT3, BIRC5 or control siRNAs for 48 hours, following which they were infected with KSHV for 3 days. Total RNA was extracted from the cells and levels of LANA and GAPDH were measured by qRT-PCR.

In RNA samples from HUVECs transfected with individual KSHV miRNAs, we observed that BIRC5 was repressed by several KSHV-encoded miRNAs (Fig. 6A). BIRC5 has been identified as a transcriptional target of STAT3^30^,^31^,^32^, but can also inhibit STAT3 function in the nucleus^33^. To identify the miRNA that might directly repress BIRC5, we performed 3′UTR luciferase reporter assays using the vector pDEST-BIRC5 and KSHV miRNA mimics (Fig. 1A, last row; Fig. 6B). Luciferase assays revealed miR-K12-5 as a miRNA that repressed the BIRC5 3′UTR by ~40% relative to the miR-Neg control (Fig. 6B). A search for potential KSHV miRNA binding sites using miRanda revealed a 7-mer binding site for miR-K12-5 in the 3′UTR of BIRC5 (Fig. 6C). We mutated this site to generate pDEST-BIRC5-mut (Fig. 6C, underlined). Luciferase reporter assays performed with pDEST-BIRC5-mut and miR-12-5 did not find repression of the mutant reporter, suggesting that this site might be important for BIRC5 repression.

To understand the contribution of the various network proteins to KSHV infection, we knocked down individual target genes in HUVECs using siRNAs, performed *de novo* infections with KSHV and measured LANA mRNA levels. BIRC5 knockdown in HUVECs prior to infection increased LANA levels by ~2-fold, while STAT3 knockdown had no effect on *de novo* infection (Fig. 6D). In HUVECs, knockdown of STAT3 with siRNAs had no effect on the levels of BIRC5, suggesting that this pathway is either not functional in primary endothelial cells or needs a specific cytokine-mediated stimulus. Together, these data may suggest that repression of BIRC5 influences KSHV infection.

### Inhibition of STAT3 in latently infected cells induces KSHV lytic reactivation

**Figure 7:**
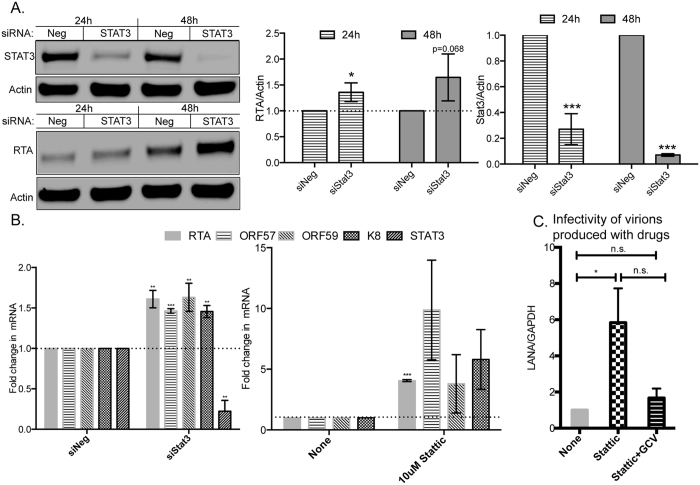
STAT3 repression in BCBL-1 cells facilitates entry into the lytic phase of the KSHV life cycle. (**A**) BCBL-1 cells were electroporated with siRNAs targeting STAT3 or control siRNA for 24 or 48 hours, following which the cells were harvested for Western blot analysis (**A**) or qRT-PCR (**B**). (**A**) The protein levels of STAT3 (top) or RTA (bottom) were normalized to that of actin and plotted on the right. B. qRT-PCR was performed 24 hours post-electroporation of siSTAT3 (left) or Stattic-treatment (right) and levels of viral lytic mRNAs (RTA, ORF57, ORF59 or K8) and STAT3 were measured by qRT-PCR. GAPDH was used as an internal control. (**C**) KSHV virions were purified from BCBL-1 cells left untreated, treated with Stattic or Stattic+GCV and then used to infect HUVECs for 3 days. LANA mRNA levels in infected HUVECs were measured using qRT-PCR, normalized to that of GAPDH, and analyzed by ANOVA. Full-length blots are in Figure S3.

In order to study the effects of STAT3 repression in the context of lytic replication, we shifted studies from endothelial cells to PEL cells, since they are a better system to study lytic replication. We reduced STAT3 levels in BCBL-1 cells using siRNAs and measured the levels of the KSHV lytic switch protein, ORF50 or RTA. Strong repression of STAT3 protein levels was observed in BCBL-1 cells at both 24 h and 48 h post-electroporation. Interestingly, in these cells, RTA protein levels were also upregulated at both time points (Fig. 7A). We also measured the mRNA levels of RTA and STAT3 using qRT-PCR and observed similar increases in the levels of RTA upon STAT3 repression. Further, we measured the levels of lytic mRNAs of KSHV, including ORF57, ORF59 and K8 and observed similar increases in their levels upon STAT3 repression (Fig. 7B). It is important to note that these increases in lytic mRNAs occurred in the absence of any chemical inducers of lytic reactivation and hence, were solely mediated by STAT3 knockdown in BCBL-1 cells.

We also inhibited STAT3 function using the STAT3 inhibitor, Stattic^34^ and observed a 5-fold increase in the mRNA levels of RTA. Similar increases in were also observed in the levels of other lytic markers like ORF57, ORF59 and K8 (Fig. 7B). Antivirals like ganciclovir (GCV) are phosphorylated to GCV-monophosphate by the late lytic proteins- ORF21 and ORF36 of KSHV, following which they can inhibit viral DNA polymerases and therefore, viral replication. However, since KSHV remains latent in a majority of PEL cells, these cells are not sensitive to GCV. In contrast, BCBL-1 cells treated with Stattic would be more sensitive to GCV due to an increase in lytic replication and the concomitant increase in viral kinases that phosphorylate GCV. We hypothesized that if we could stimulate latently infected BCBL-1 cells to reactivate with Stattic, then virion production could be inhibited using GCV. To test this hypothesis, we treated BCBL-1 cells with Stattic or a combination of Stattic and GCV. Virions from the cell-free supernatants were harvested two days post-treatment and used to infect HUVECs to determine viral loads via qRT-PCR for LANA mRNA. Consistent with Fig. 7B, we observed almost a 6-fold increase in virus production in the presence of Stattic when compared to DMSO-treated control cells (Fig. 7C). Drug-treatment of BCBL-1 cells with Stattic did not severely affect cell viability (decrease of viability with Stattic was 6.25% +/− 0.5%, by trypan blue assay) and therefore, the increase in virus production is not likely due to cell death. Virus production with a combination of Stattic and ganciclovir was similar to uninduced cells, suggesting that ganciclovir inhibited virus production.

## Discussion

We report a protein network centering on STAT3 that is targeted by KSHV-encoded miRNAs to inhibit the action of cytokines, suppress interferon responses and regulate lytic replication. The different members in this network are individually repressed by KSHV miRNAs using both canonical and non-canonical base-pairing interactions in their 3′UTRs. By suppressing the core member of this network- STAT3, KSHV miRNAs are able to inhibit a multitude of pathways involving cytokines like IL6, EPO and IFN. Further, we demonstrate that STAT3 repression (by siRNAs or with drugs) can trigger KSHV lytic replication in PEL cells, as a result of which they become more sensitive to antivirals like ganciclovir.

IFNs play an important role in the establishment of innate and adaptive immunity against viruses. IFNs and the ISGs they activate are widely considered to be tumor-suppressive and therefore, inhibition of this major antiviral pathway can prove to be beneficial for both viral persistence and tumor establishment. Consequently, many viruses including KSHV have evolved elaborate mechanisms to overcome this pathway. KSHV encodes many proteins in its genome to counteract the actions of IFNs. For instance, the viral IRF-2 protein (vIRF2) inhibits the formation of the ISGF3 (interferon stimulated gene factor 3) complex upon IFN-α engagement with its receptors. The viral IL-6 protein (vIL6) also inhibits the ISGF3 binding to DNA elements to block the synthesis of ISGs (For review^35^). In this report, we demonstrate that multiple KSHV miRNAs that repress STAT3 can weaken the innate immune responses to type-I interferons and inhibit induction of antiviral ISGs like IRF1, IFITM1 and ISG15. Among KSHV miRNAs, miR-K11 has been demonstrated to inhibit IFN signaling upon sendai viral infection by targeting IKKε^36^. Viral miRNA-mediated inhibition of IFN-responses also has precedents in other viruses. For instance, the anneloviral miRNAs were demonstrated to inhibit type-I interferon signaling and promote cell survival under IFN-rich conditions^37^. KSHV miRNAs can similarly promote spindle cell persistence under high interferon levels that are normally observed in the KS tumor. It is to be noted that vIRF and vIL6 are primarily expressed during the lytic phase; on the other hand, viral miRNAs can inhibit antiviral responses during latency.

Previously it was demonstrated that KSHV infection of endothelial cells resulted in a biphasic activation of STAT3^38^. Punjabi *et al*., observed phosphorylation of STAT3 (at Y705) upon *de novo* infection of HUVECs. However, we note that HUVECs that were transfected with siSTAT3 prior to de novo KSHV infection, had LANA levels that were comparable to those from cells transfected with control siRNA (Fig. 6D). KSHV miRNAs may facilitate waning of pY705-STAT3 levels at certain time points that was reported after *de novo* KSHV infection of endothelial cells^38^. KSHV proteins vGPCR and kaposin B have been shown to increase STAT3 phosphorylation^39^,^40^. It is possible that in latently infected cells, proper regulation of IL6 signaling and STAT3 phosphorylation is important for viral persistence. This proper regulation is likely achieved through a mixture of positive and negative regulators. A recent study reported an indirect activation of STAT3 by KSHV-encoded miRNAs (including miR-K6)^41^. Previously, we tested sixteen KSHV miRNAs in primary endothelial cells and no miRNAs increased STAT3 protein levels^15^. At the moment, our results do not have information about the role of KSHV miRNAs and STAT3 signaling in B cells. While we acknowledge the differences between these previous studies and our current work, it is essential to note that in certain genetic contexts, e.g. the absence of PTEN, STAT3 can also function as a tumor suppressor^42^. While PTEN deletions are uncommon in KSHV infections, PTEN inactivation by phosphorylation in KSHV infections have been reported^43^,^44^. STAT3 suppression when combined with PTEN inactivation might therefore accelerate cancer progression. The role of other causal mutations that occur in KSHV infection and how STAT3 repression plays into it would require further investigation. Although repression of STAT3 activation is novel for KSHV, it is not an entirely new concept. STAT3 inhibition is known to benefit many viruses. The mumps viral V protein can target STAT3 to ubiquitin-mediated degradation^45^ and the V protein of measles forms a complex with STAT proteins to prevent nuclear translocation^46^. The P protein of rabies virus associates with STAT3 in an oncostatin M- dependent manner to inhibit STAT3 activation^47^. STAT3 is also inhibited by the human cytomegalovirus (hCMV)^48^ and the human metapneumovirus (hMPV)^49^. The v-cyclin protein of KSHV interacts with STAT3 to inhibit oncostatin M- mediated growth inhibition^50^. We also demonstrate the repression of STAT5 activation by KSHV miRNAs in endothelial cells via EPOR repression. Erythropoietin (EPO), a glycoprotein hormone that is essential for erythropoiesis, acts on endothelial cells to stabilize the vascular matrix^51^. KS involves the infection of endothelial cells by KSHV, following which the proliferating ‘spindle-cells’ lead to leaky blood vessels and extravasation of red blood cells at the site of the lesion. EPOR repression in endothelial cells might contribute to the loss of vascular architecture that is characteristic of KS. Interestingly, one of the targets of EPO in endothelial cells is thrombospondin 1 (THBS1), an antiangiogenic factor that is a target of KSHV miRNAs^14^. Therefore, loss of EPOR expression in endothelial cells can accelerate loss of THBS1 expression due to KSHV miRNAs. Human CMV, a β-herpesvirus, was shown to reduce erythropoietin levels under hypoxic conditions^52^. The observation that KSHV can repress EPOR-dependent STAT5 activation is novel and highlights the differences in the mechanisms adopted by related herpesviruses to inhibit the EPO pathway. We also identify and validate BIRC5 as a novel target of KSHV miRNAs and our data suggests that BIRC5 repression may increase the susceptibility of endothelial cells to KSHV infection (Fig. 6). KSHV miRNAs have been demonstrated to be present in exosomes^53^. It is possible that that these exosomal miRNAs, upon uptake by a target cell, repress levels of BIRC5, which then increases its susceptibility to subsequent KSHV infections.

Finally in PEL cells, inhibition of STAT3 activity promoted entry into the lytic phase of the KSHV life cycle. Our results are supported by prior studies from a closely related γ-herpesvirus, Epstein-Barr virus (EBV), where it was reported that high levels of STAT3 in EBV-infected cells were refractory to lytic reactivation, while STAT3 levels were reduced in lytic cells^54^,^55^. Treatment of EBV-infected cells with STAT3 inhibitors also promoted lytic reactivation^55^. Interestingly, inhibition of STAT3 activation promoted HIV-1 Tat-dependent KSHV lytic reactivation^56^, supporting our observations that STAT3 may function as an inhibitor of the lytic cycle. A recent study showed that inhibition of STAT3 in PEL cells enhances KSHV lytic replication and correlates with our findings^57^. We have also extended this to further demonstrate a potential strategy for elimination of latent cells using a combination of a STAT3 inhibitor and ganciclovir (Fig. 7C). It is also possible that modest regulation of STAT3 expression or activation during latency may contribute to the low level of basal lytic replication observed in KSHV infected B-cells. Patients with autosomal dominant hyper-IgE syndrome (AD-HIES or Job’s syndrome) have mutations in their *STAT3* gene and this reduces the levels of functional STAT3 protein^58^,^59^. EBV-infected cells from AD-HIES patients also have a higher degree of spontaneous lytic reactivation^55^. AD-HIES patients have a higher risk for VZV (varicella zoster virus) reactivation and increased EBV viremia^60^. Lytic reactivation of KSHV with valproate in the presence of antivirals like ganciclovir has shown to induce PEL cell apoptosis^61^. In summary, we have identified several novel targets of KSHV miRNAs and demonstrated how viral miRNAs could regulate responses to cellular stimuli.

## Experimental Methods

### Cell culture and reagents

Human umbilical vein endothelial cells (HUVECs; Lonza, Walkersville, MD) were maintained for five passages in complete EGM-2 BulletKit (Lonza). The KSHV-latently infected cell line, BCBL-1, was maintained in RPMI 1640 supplemented with 10% FBS, 1X Peniciliin-Streptomycin and 55 μM β-mercaptoethanol. 293 cells were maintained in DMEM supplemented with 10% FBS and 1X Penicillin-Streptomycin. miRVana miRNA mimics of various KSHV-encoded miRNAs were purchased from Ambion (Life Technologies Inc., Carlsbad, CA). ON-TARGETplus SMARTpool siRNAs targeting human STAT3, BIRC5, IRAK1 and the non-targeting pool control were obtained from Thermo Fisher Scientific (Waltham, MA). The STAT3 inhibitor- Stattic, was purchased from Sigma-Aldrich Inc, St. Louis, MO. The STAT3-dependent luciferase expression plasmid, 4xM67 pTATA TK-Luc, was a gift from Jim Darnell and was obtained through Addgene.

### miRNA transfections and Western blotting

miRNA transfections of HUVECs were performed with 10 nM miRNAs or 16.5 nM siRNAs using Dharmafect (Thermo Fisher Scientific). The transfected cells were harvested 48-hours post-transfection (hpt) and their total protein extracted in radio-immunoprecipitation assay buffer (RIPA; Sigma) containing 1x Halt Protease and Phosphatase inhibitors (Thermo Fisher Scientific). The LI-COR Odyssey Infrared Imaging System (LI-COR Biosciences, Lincoln, NE) was utilized for measurement of protein levels. Primary antibodies were obtained from Cell Signaling Technology Inc., Danvers, MA, unless specified otherwise. Mouse-anti-actin primary antibody (AC-74) was obtained from Sigma. The rabbit anti-RTA antibody was a gift from Don Ganem. Secondary antibodies conjugated to infrared fluorescing dyes- IRDye 800 CW and IRDye 680, were obtained from LI-COR. Changes in protein levels were measured relative to actin.

### 3′UTR luciferase assays

3′UTR-dual luciferase reporter assays were performed in 293 cells as reported previously^23^. Briefly, 3′UTRs of various genes were cloned downstream of renilla luciferase gene in the vector, pDEST-765 (Protein Expression Laboratory, Leidos, Frederick, MD) and the HSV-TK-promoter driven firefly luciferase served as an internal control. Upon cotransfection of 293 cells with pDEST-765-3′UTR and KSHV miRNA mimics, we normalized the renilla luciferase values to the internal firefly luciferase control reporter, a parental control reporter lacking cloned 3′UTRs, and a non-targeting negative control miRNA mimic. Cells were harvested 24 or 48 hpt and dual luciferase reporter assays were performed using the Dual-Luciferase Reporter system (Promega, Madison, WI). Mutations of miRNA-binding sites in 3′UTRs were performed using the QuikChange II Site-Directed Mutagenesis kit (Stratagene, San Diego, CA).

### Cytokine treatments and measurement of phospho-STAT3

HUVECs were transfected with miRNAs or siRNAs as described above. Forty-eight hours after transfection, the cells were treated with 10 ng/ml of IL6 (Cell Signaling Technology) or 1000 U/ml of IFNαA (Sigma) for 20 minutes after which cells were washed once with ice-cold PBS and harvested in RIPA buffer. Erythropoietin treatments of HUVECs were performed with 250 U of EPO (Sigma) for 10 minutes. Equal amount of lysates were used for Western blot analysis to measure levels of pY705-STAT3, pS727-STAT3, STAT3, PKCδ, IRAK1, pY701-STAT1, pY694-STAT5 or STAT5 and their levels normalized to that of actin. When necessary, blots were stripped of their primary antibody using the Newblot Nitro Stripping buffer (Li-COR) and reprobed.

### STAT3 activation luciferase reporter assays

293 cells were reverse-transfected with KSHV miRNAs, the STAT3-reporter plasmid- 4xM67 pTATA TK-Luc and a control renilla luciferase expression plasmid (pRNL) using Lipofectamine 2000 (Life Technologies). Sixteen hours post-transfection, media was changed to that containing 20 ng/ml IL6. Cells were harvested eight hours post-IL6 addition and luciferase assays were performed as described earlier. Fold activation in response to IL6 addition were calculated relative to untreated controls.

### KSHV purification and infection

KSHV production was induced in BCBL-1 cells using 1 mM valproic acid for seven days. The Vivaflow 50 tangential flow filtration system (Sartorius Stedim Biotech, Goettingen, Germany) was used to concentrate the virions released into the culture supernatants. KSHV infection of HUVECs were performed in media containing 8 μg/ml polybrene for four hours at 37 °C. Media was changed every two days and cells were harvested for analysis by Western blotting or real-time quantitative PCR (qRT-PCR).

### Real-time quantitative PCR analysis

Total RNA was extracted using the miRNeasy mini kit (Qiagen) and converted to cDNA using the High Capacity cDNA Reverse Transcription kit (Applied Biosystems, Foster City, CA). The following primers were used for measurement of LANA mRNA levels: 5′-GTGACCTTGGCGATGACCTA-3′ and 5′-CAGGAGATGGAGAATGAGTA-3′. STAT3 mRNA levels were measured using 5′-GGTGTCTCCACTGGTCTATC-3′ and 5′-GGTATTGCTGCAGGTCGT-3′ primers. KSHV lytic mRNAs were measured using primer pairs for RTA, ORF57, ORF59 and K8 as described^62^. GAPDH mRNA was used as a normalization control. qPCR analysis was performed using FastStart Universal SYBR Green Master (ROX) (Roche Diagnostics GmbH). Taqman Gene Expression Assays targeting cellular genes were purchased from Applied Biosystems.

### BCBL-1 electroporations and measure of lytic replication

Electroporation of siRNAs into BCBL-1 cells were performed using the Amaxa 4D-Nucleofector (Lonza) with CA-189 program in the Nucleofector buffer SF following manufacturer’s instructions. Cells were harvested 24 hours post-electroporation and the levels of STAT3 and KSHV lytic mRNAs measured using qRT-PCR. BCBL-1 cells were also treated with the STAT3 inhibitor- Stattic or DMSO as control, for 24 hours and the levels of STAT3 and lytic mRNAs were measured using qRT-PCR. For assessment of viral loads upon drug treatment, BCBL-1 cells were treated with Stattic or ganciclovir for 2 days, following which the viruses in the cell-free supernatants were used to infect HUVECs as described earlier. RNA was extracted from infected HUVECs after three days and LANA mRNA levels were quantified using qRT-PCR.

### Network Analysis

Luciferase validated miRNA targets (34 in total, 28 from microarrays, 6 from proteomic screen) were input into the MetaCore analysis software (Thomson Reuters). The network was generated using the direct interactions algorithm. This created a network of only the 34 targets and their direct interactions based on the database of interactions in the MetaCore software.

### Statistical significance

Statistical significance relative to corresponding control samples were analyzed using a Student’s t-test. Significance values of p < 0.05 are denoted by *p < 0.001 by and **p < 0.0001 by ***unless otherwise indicated. In some cases, ANOVA was used instead of t-tests.

### Data Reporting

Expression data can be retrieved from NCBI GEO using accession numbers GSE12967, GSE40093, GSE24069, GSE65148.

## Additional Information

**How to cite this article:** Ramalingam, D. and Ziegelbauer, J. M. Viral microRNAs Target a Gene Network, Inhibit STAT Activation, and Suppress Interferon Responses. *Sci. Rep.* **7**, 40813; doi: 10.1038/srep40813 (2017).

**Publisher's note:** Springer Nature remains neutral with regard to jurisdictional claims in published maps and institutional affiliations.

## Supplementary Material

Supplementary Information

## References

[b1] Pfeffer S. . Identification of microRNAs of the herpesvirus family. Nat Methods 2, 269–276 (2005). 15782219 10.1038/nmeth746

[b2] Cai X. . Kaposi’s sarcoma-associated herpesvirus expresses an array of viral microRNAs in latently infected cells. Proc Natl Acad Sci USA 102, 5570–5575 (2005). 15800047 10.1073/pnas.0408192102 PMC556237

[b3] Samols M. A., Hu J., Skalsky R. L. & Renne R. Cloning and identification of a microRNA cluster within the latency-associated region of Kaposi’s sarcoma-associated herpesvirus. J Virol 79, 9301–9305 (2005). 15994824 10.1128/JVI.79.14.9301-9305.2005 PMC1168752

[b4] Fabian M. R., Sonenberg N. & Filipowicz W. Regulation of mRNA translation and stability by microRNAs. Annu Rev Biochem 79, 351–379 (2010). 20533884 10.1146/annurev-biochem-060308-103103

[b5] Loeb G. B. . Transcriptome-wide miR-155 binding map reveals widespread noncanonical microRNA targeting. Mol Cell 48, 760–770 (2012). 23142080 10.1016/j.molcel.2012.10.002 PMC3562697

[b6] Chi S. W., Hannon G. J. & Darnell R. B. An alternative mode of microRNA target recognition. Nat Struct Mol Biol 19, 321–327 (2012). 22343717 10.1038/nsmb.2230 PMC3541676

[b7] Gottwein E. . Viral MicroRNA Targetome of KSHV-Infected Primary Effusion Lymphoma Cell Lines. Cell Host Microbe 10, 515–526 (2011). 22100165 10.1016/j.chom.2011.09.012 PMC3222872

[b8] Ramalingam D., Kieffer-Kwon P. & Ziegelbauer J. M. Emerging themes from EBV and KSHV microRNA targets. Viruses 4, 1687–1710 (2012). 23170179 10.3390/v4091687 PMC3499826

[b9] Boss I. W., Plaisance K. B. & Renne R. Role of virus-encoded microRNAs in herpesvirus biology. Trends Microbiol 17, 544–553 (2009). 19828316 10.1016/j.tim.2009.09.002 PMC2802859

[b10] Qin Z., Jakymiw A., Findlay V. & Parsons C. KSHV-Encoded MicroRNAs: Lessons for Viral Cancer Pathogenesis and Emerging Concepts. Int J Cell Biol 2012, 603961, doi: 10.1155/2012/603961 (2012). 22505930 PMC3296157

[b11] Gottwein E. . A viral microRNA functions as an orthologue of cellular miR-155. Nature 450, 1096–1099 (2007). 18075594 10.1038/nature05992 PMC2614920

[b12] Nachmani D., Stern-Ginossar N., Sarid R. & Mandelboim O. Diverse herpesvirus microRNAs target the stress-induced immune ligand MICB to escape recognition by natural killer cells. Cell Host Microbe 5, 376–385 (2009). 19380116 10.1016/j.chom.2009.03.003

[b13] Ziegelbauer J. M., Sullivan C. S. & Ganem D. Tandem array-based expression screens identify host mRNA targets of virus-encoded microRNAs. Nat Genet 41, 130–134 (2009). 19098914 10.1038/ng.266 PMC2749995

[b14] Samols M. A. . Identification of cellular genes targeted by KSHV-encoded microRNAs. PLoS Pathog 3, e65, doi: 10.1371/journal.ppat.0030065 (2007). 17500590 PMC1876501

[b15] Gallaher A. M. . Proteomic screening of human targets of viral microRNAs reveals functions associated with immune evasion and angiogenesis. PLoS Pathog 9, e1003584, doi: 10.1371/journal.ppat.1003584 (2013). 24039573 PMC3764211

[b16] Haecker I. . Ago HITS-CLIP expands understanding of Kaposi’s sarcoma-associated herpesvirus miRNA function in primary effusion lymphomas. PLoS Pathog 8, e1002884, doi: 10.1371/journal.ppat.1002884 (2012). 22927820 PMC3426530

[b17] Skalsky R. L. . The viral and cellular microRNA targetome in lymphoblastoid cell lines. PLoS Pathog 8, e1002484, doi: 10.1371/journal.ppat.1002484 (2012). 22291592 PMC3266933

[b18] Jain N., Zhang T., Kee W. H., Li W. & Cao X. Protein kinase C delta associates with and phosphorylates Stat3 in an interleukin-6-dependent manner. J Biol Chem 274, 24392–24400 (1999). 10446219 10.1074/jbc.274.34.24392

[b19] Huang Y., Li T., Sane D. C. & Li L. IRAK1 serves as a novel regulator essential for lipopolysaccharide-induced interleukin-10 gene expression. J Biol Chem 279, 51697–51703 (2004). 15465816 10.1074/jbc.M410369200

[b20] Platanias L. C. Mechanisms of type-I- and type-II-interferon-mediated signalling. Nat Rev Immunol 5, 375–386 (2005). 15864272 10.1038/nri1604

[b21] Abend J. R. . Kaposi’s sarcoma-associated herpesvirus microRNAs target IRAK1 and MYD88, two components of the toll-like receptor/interleukin-1R signaling cascade, to reduce inflammatory-cytokine expression. J Virol 86, 11663–11674 (2012). 22896623 10.1128/JVI.01147-12 PMC3486292

[b22] Kieffer-Kwon P., Happel C., Uldrick T. S., Ramalingam D. & Ziegelbauer J. M. KSHV MicroRNAs Repress Tropomyosin 1 and Increase Anchorage-Independent Growth and Endothelial Tube Formation. PLoS One 10, e0135560, doi: 10.1371/journal.pone.0135560 (2015). 26263384 PMC4532463

[b23] Abend J. R., Uldrick T. & Ziegelbauer J. M. Regulation of tumor necrosis factor-like weak inducer of apoptosis receptor protein (TWEAKR) expression by Kaposi’s sarcoma-associated herpesvirus microRNA prevents TWEAK-induced apoptosis and inflammatory cytokine expression. J Virol 84, 12139–12151 (2010). 20844036 10.1128/JVI.00884-10 PMC2976403

[b24] Lewis B. P., Burge C. B. & Bartel D. P. Conserved seed pairing, often flanked by adenosines, indicates that thousands of human genes are microRNA targets. Cell 120, 15–20 (2005). 15652477 10.1016/j.cell.2004.12.035

[b25] Enright A. J. . MicroRNA targets in Drosophila. Genome Biol 5, R1, doi: 10.1186/gb-2003-5-1-r1 (2003). 14709173 PMC395733

[b26] Thomson D. W., Bracken C. P., Szubert J. M. & Goodall G. J. On measuring miRNAs after transient transfection of mimics or antisense inhibitors. PLoS One 8, e55214, doi: 10.1371/journal.pone.0055214 (2013). 23358900 PMC3554668

[b27] Ho H. H. & Ivashkiv L. B. Role of STAT3 in type I interferon responses. Negative regulation of STAT1-dependent inflammatory gene activation. J Biol Chem 281, 14111–14118 (2006). 16571725 10.1074/jbc.M511797200

[b28] Schoggins J. W. Interferon-stimulated genes: roles in viral pathogenesis. Curr Opin Virol 6, 40–46 (2014). 24713352 10.1016/j.coviro.2014.03.006 PMC4077717

[b29] Kirito K., Uchida M., Yamada M., Miura Y. & Komatsu N. A distinct function of STAT proteins in erythropoietin signal transduction. J Biol Chem 272, 16507–16513 (1997). 9195960 10.1074/jbc.272.26.16507

[b30] Aoki Y., Feldman G. M. & Tosato G. Inhibition of STAT3 signaling induces apoptosis and decreases survivin expression in primary effusion lymphoma. Blood 101, 1535–1542 (2003). 12393476 10.1182/blood-2002-07-2130

[b31] Gritsko T. . Persistent activation of stat3 signaling induces survivin gene expression and confers resistance to apoptosis in human breast cancer cells. Clin Cancer Res 12, 11–19 (2006). 16397018 10.1158/1078-0432.CCR-04-1752

[b32] Jiang H., Yu J., Guo H., Song H. & Chen S. Upregulation of survivin by leptin/STAT3 signaling in MCF-7 cells. Biochem Biophys Res Commun 368, 1–5 (2008). 18242580 10.1016/j.bbrc.2007.04.004

[b33] Wang H. . Acetylation directs survivin nuclear localization to repress STAT3 oncogenic activity. J Biol Chem 285, 36129–36137 (2010). 20826784 10.1074/jbc.M110.152777 PMC2975235

[b34] Schust J., Sperl B., Hollis A., Mayer T. U. & Berg T. Stattic: a small-molecule inhibitor of STAT3 activation and dimerization. Chem Biol 13, 1235–1242 (2006). 17114005 10.1016/j.chembiol.2006.09.018

[b35] Areste C. & Blackbourn D. J. Modulation of the immune system by Kaposi’s sarcoma-associated herpesvirus. Trends Microbiol 17, 119–129 (2009). 19230674 10.1016/j.tim.2008.12.001

[b36] Liang D. . A human herpesvirus miRNA attenuates interferon signaling and contributes to maintenance of viral latency by targeting IKKepsilon. Cell research 21, 793–806 (2011). 21221132 10.1038/cr.2011.5 PMC3325102

[b37] Kincaid R. P., Burke J. M., Cox J. C., de Villiers E. M. & Sullivan C. S. A human torque teno virus encodes a microRNA that inhibits interferon signaling. PLoS Pathog 9, e1003818, doi: 10.1371/journal.ppat.1003818 (2013). 24367263 PMC3868544

[b38] Punjabi A. S., Carroll P. A., Chen L. & Lagunoff M. Persistent activation of STAT3 by latent Kaposi’s sarcoma-associated herpesvirus infection of endothelial cells. J Virol 81, 2449–2458 (2007). 17151100 10.1128/JVI.01769-06 PMC1865938

[b39] Burger M., Hartmann T., Burger J. A. & Schraufstatter I. KSHV-GPCR and CXCR2 transforming capacity and angiogenic responses are mediated through a JAK2-STAT3-dependent pathway. Oncogene 24, 2067–2075 (2005). 15688008 10.1038/sj.onc.1208442

[b40] King C. A. Kaposi’s sarcoma-associated herpesvirus kaposin B induces unique monophosphorylation of STAT3 at serine 727 and MK2-mediated inactivation of the STAT3 transcriptional repressor TRIM28. J Virol 87, 8779–8791 (2013). 23740979 10.1128/JVI.02976-12 PMC3719813

[b41] Li W. . The SH3BGR/STAT3 Pathway Regulates Cell Migration and Angiogenesis Induced by a Gammaherpesvirus MicroRNA. PLoS Pathog 12, e1005605, doi: 10.1371/journal.ppat.1005605 (2016). 27128969 PMC4851422

[b42] Pencik J. . STAT3 regulated ARF expression suppresses prostate cancer metastasis. Nature communications 6, 7736, doi: 10.1038/ncomms8736 (2015). PMC4525303 26198641

[b43] Roy D. & Dittmer D. P. Phosphatase and tensin homolog on chromosome 10 is phosphorylated in primary effusion lymphoma and Kaposi’s sarcoma. The American journal of pathology 179, 2108–2119 (2011). 21819957 10.1016/j.ajpath.2011.06.017 PMC3181371

[b44] Tomlinson C. C. & Damania B. The K1 protein of Kaposi’s sarcoma-associated herpesvirus activates the Akt signaling pathway. Journal of virology 78, 1918–1927 (2004). 14747556 10.1128/JVI.78.4.1918-1927.2004 PMC369501

[b45] Ulane C. M., Rodriguez J. J., Parisien J. P. & Horvath C. M. STAT3 ubiquitylation and degradation by mumps virus suppress cytokine and oncogene signaling. J Virol 77, 6385–6393 (2003). 12743296 10.1128/JVI.77.11.6385-6393.2003 PMC155014

[b46] Palosaari H., Parisien J. P., Rodriguez J. J., Ulane C. M. & Horvath C. M. STAT protein interference and suppression of cytokine signal transduction by measles virus V protein. J Virol 77, 7635–7644 (2003). 12805463 10.1128/JVI.77.13.7635-7644.2003 PMC164804

[b47] Lieu K. G. . The rabies virus interferon antagonist P protein interacts with activated STAT3 and inhibits Gp130 receptor signaling. J Virol 87, 8261–8265 (2013). 23698294 10.1128/JVI.00989-13 PMC3700209

[b48] Reitsma J. M., Sato H., Nevels M., Terhune S. S. & Paulus C. Human cytomegalovirus IE1 protein disrupts interleukin-6 signaling by sequestering STAT3 in the nucleus. J Virol 87, 10763–10776 (2013). 23903834 10.1128/JVI.01197-13 PMC3807375

[b49] Mitzel D. N., Jaramillo R. J., Stout-Delgado H., Senft A. P. & Harrod K. S. Human metapneumovirus inhibits the IL-6-induced JAK/STAT3 signalling cascade in airway epithelium. J Gen Virol 95, 26–37 (2014). 24114793 10.1099/vir.0.055632-0 PMC5974310

[b50] Lundquist A. . Kaposi sarcoma-associated viral cyclin K overrides cell growth inhibition mediated by oncostatin M through STAT3 inhibition. Blood 101, 4070–4077 (2003). 12531804 10.1182/blood-2002-07-1994

[b51] Fliser D. & Bahlmann F. H. Erythropoietin and the endothelium–a promising link? Eur J Clin Invest 38, 457–461 (2008). 18505404 10.1111/j.1365-2362.2008.01968.x

[b52] Butler L. M. . Human cytomegalovirus inhibits erythropoietin production. J Am Soc Nephrol 25, 1669–1678 (2014). 24722450 10.1681/ASN.2013101125 PMC4116070

[b53] Chugh P. E. . Systemically circulating viral and tumor-derived microRNAs in KSHV-associated malignancies. PLoS Pathog 9, e1003484, doi: 10.1371/journal.ppat.1003484 (2013). 23874201 PMC3715412

[b54] Daigle D. . Upregulation of STAT3 marks Burkitt lymphoma cells refractory to Epstein-Barr virus lytic cycle induction by HDAC inhibitors. J Virol 84, 993–1004 (2010). 19889776 10.1128/JVI.01745-09 PMC2798381

[b55] Hill E. R. . Signal transducer and activator of transcription 3 limits Epstein-Barr virus lytic activation in B lymphocytes. J Virol 87, 11438–11446 (2013). 23966384 10.1128/JVI.01762-13 PMC3807321

[b56] Zeng Y. . Intracellular Tat of human immunodeficiency virus type 1 activates lytic cycle replication of Kaposi’s sarcoma-associated herpesvirus: role of JAK/STAT signaling. J Virol 81, 2401–2417 (2007). 17151125 10.1128/JVI.02024-06 PMC1865948

[b57] King C. A., Li X., Barbachano-Guerrero A. & Bhaduri-McIntosh S. STAT3 Regulates Lytic Activation of Kaposi’s Sarcoma-Associated Herpesvirus. J Virol 89, 11347–11355 (2015). 26339061 10.1128/JVI.02008-15 PMC4645641

[b58] Minegishi Y. . Dominant-negative mutations in the DNA-binding domain of STAT3 cause hyper-IgE syndrome. Nature 448, 1058–1062 (2007). 17676033 10.1038/nature06096

[b59] Holland S. M. . STAT3 mutations in the hyper-IgE syndrome. The New England journal of medicine 357, 1608–1619 (2007). 17881745 10.1056/NEJMoa073687

[b60] Siegel A. M. . A critical role for STAT3 transcription factor signaling in the development and maintenance of human T cell memory. Immunity 35, 806–818 (2011). 22118528 10.1016/j.immuni.2011.09.016 PMC3228524

[b61] Klass C. M., Krug L. T., Pozharskaya V. P. & Offermann M. K. The targeting of primary effusion lymphoma cells for apoptosis by inducing lytic replication of human herpesvirus 8 while blocking virus production. Blood 105, 4028–4034 (2005). 15687238 10.1182/blood-2004-09-3569 PMC1895088

[b62] Scholz B. A. . Abortive lytic reactivation of KSHV in CBF1/CSL deficient human B cell lines. PLoS Pathog 9, e1003336, doi: 10.1371/journal.ppat.1003336 (2013). 23696732 PMC3656114

