# Supplementary material for: Viral microRNAs Target a Gene Network, Inhibit STAT Activation, and Suppress Interferon Responses
Source: Sci Rep. 2017 Jan 19;7:40813. doi: 10.1038/srep40813 (PMC5244407; doi:10.1038/srep40813)
Supplement: Supplementary Information [file srep40813-s1.pdf]

Viral microRNAs Target a Gene Network, Inhibit STAT Activation, and Suppress Interferon Responses

Dhivya Ramalingam[1] and Joseph M. Ziegelbauer[1*]

[1] HIV and AIDS Malignancy Branch, National Cancer Institute, National Institutes of Health, Bethesda, Maryland 20892.

Supplementary Information

Figure S1. Phosphorylated and total STAT information.

Figure S2. Full-length blots for Figure 3.

Figure S3. Full-length blots for Figures 4, 5, and 7.

Table S1. Luciferase data is shown (mean and standard deviation, n = 3) for all tested combinations of 3'UTR luciferase reporters and KSHV miRNAs reported in Figure 1.

Table S2. Sequences of the oligonucleotide primers used for the identification of non-canonical binding sites in the 3' untranslated regions of KSHV miRNA target genes reported in Figure 1.

Table S3. Interactions report from MetaCore analysis lists the publications that each interaction is based upon in Figure 2.

Table S4. BIRC5 3'UTR luciferase data.

Figure S1

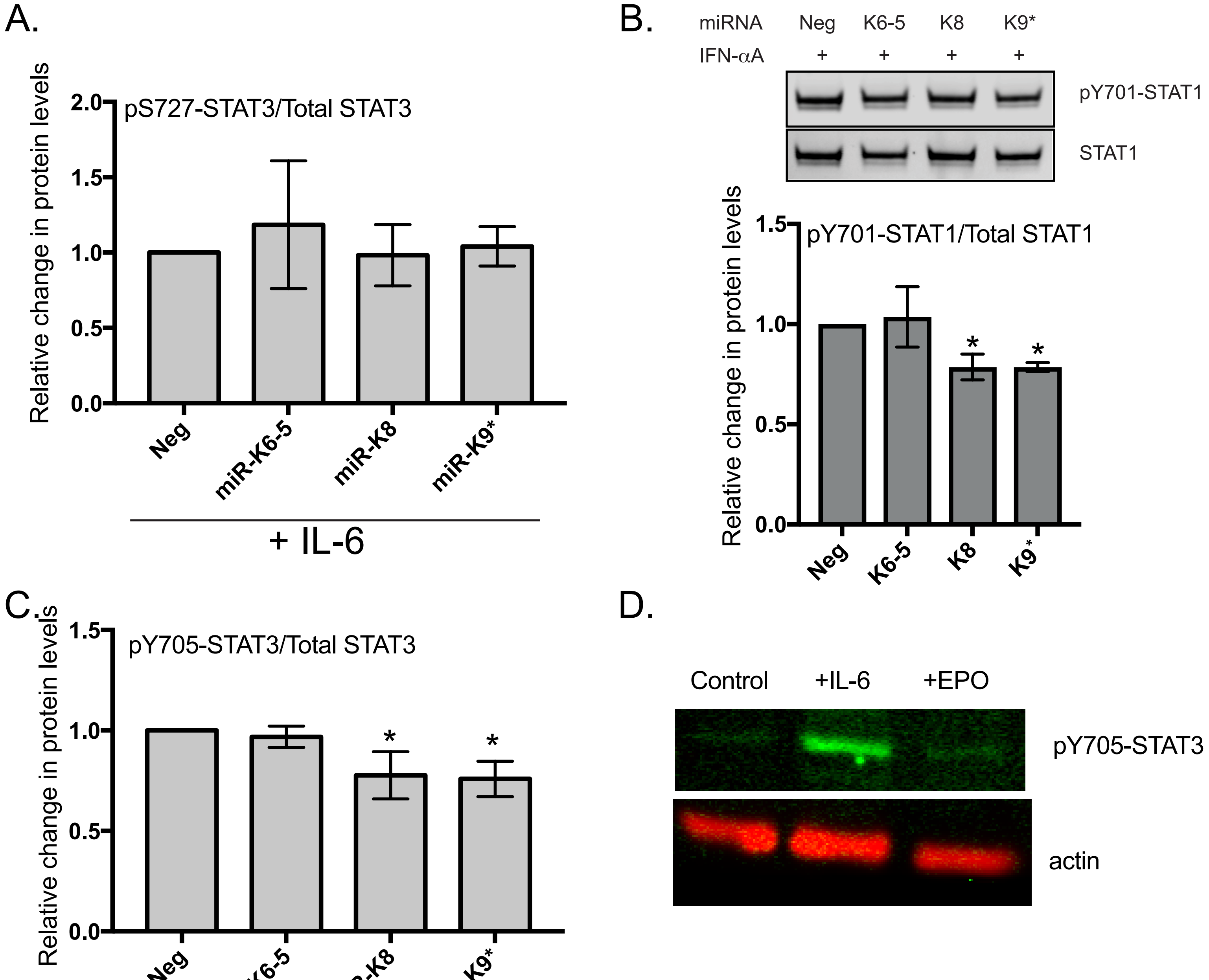

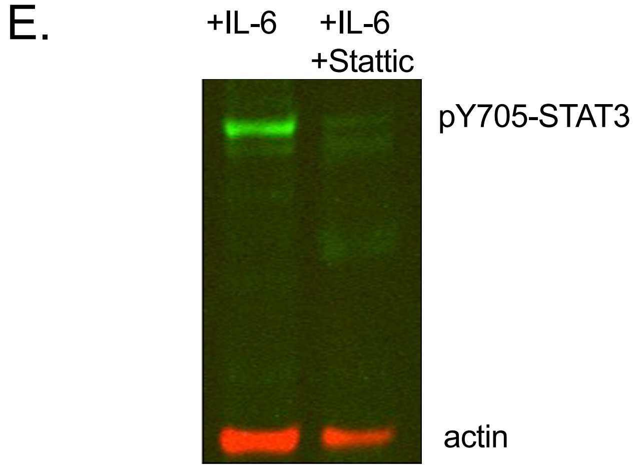

Supplementary Figure S1. Analysis of phosphorylated STAT proteins.
A. Ratios of pS727-STAT3 to total STAT3 are shown for IL-6 treated HUVECs. B. Western blot and ratios of pY701-STAT1 to total STAT1 are shown for IFN-aA treated HUVECs. C. Ratios of pY705-STAT3 to total STAT3 are shown for IFN-aA treated HUVECs. D. Western blot for IL-6 and EPO treated HUVECs are shown. E. Western blot for BCBL-1 cells treated with IL-6 and Stattic are shown.

Figure S2

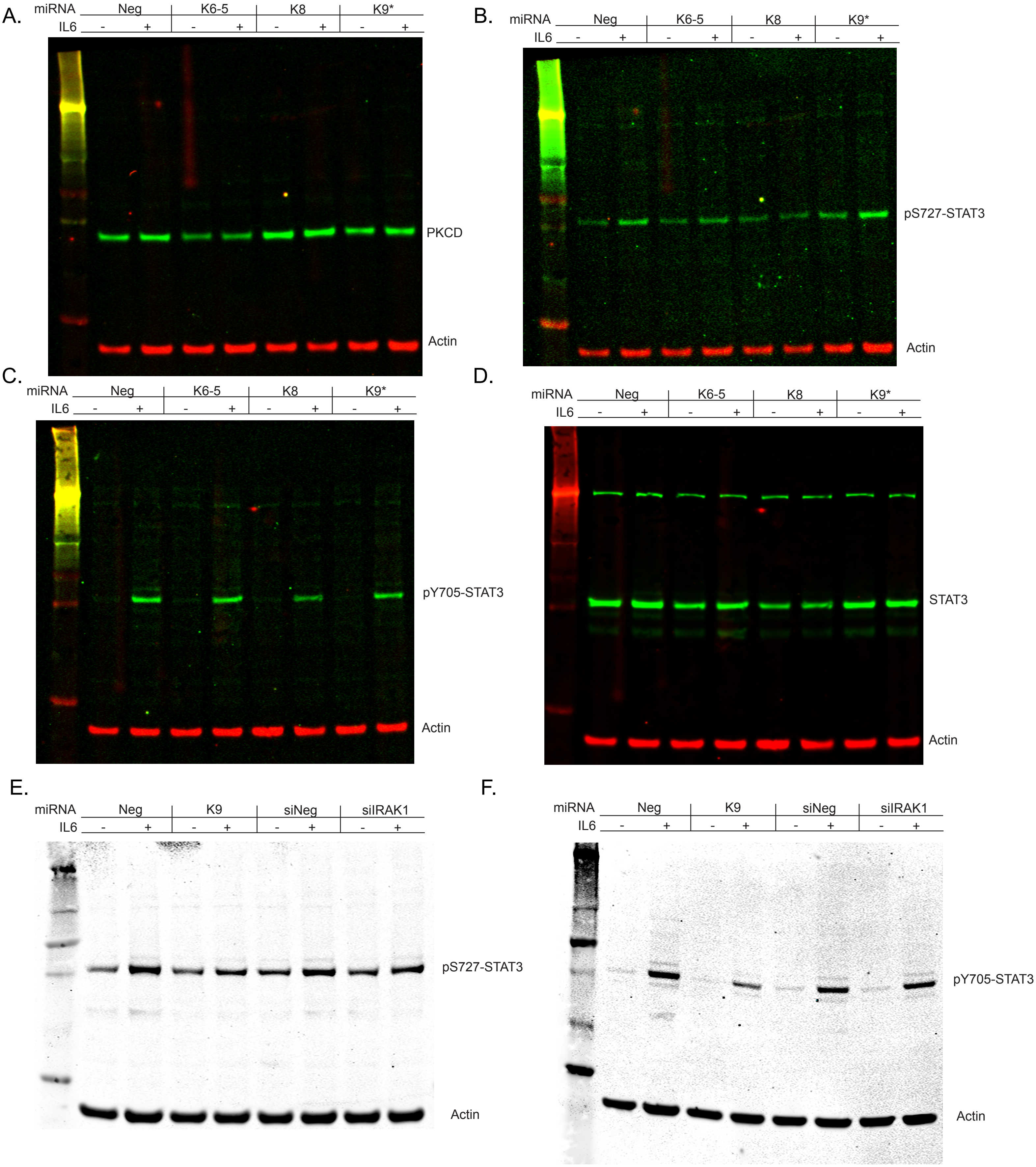

Supplementary Figure S2. Immunoblots in main figures.
A-D. Full blots for Figure 3A. E-F. Full blots for Figure 3B.

Figure S3

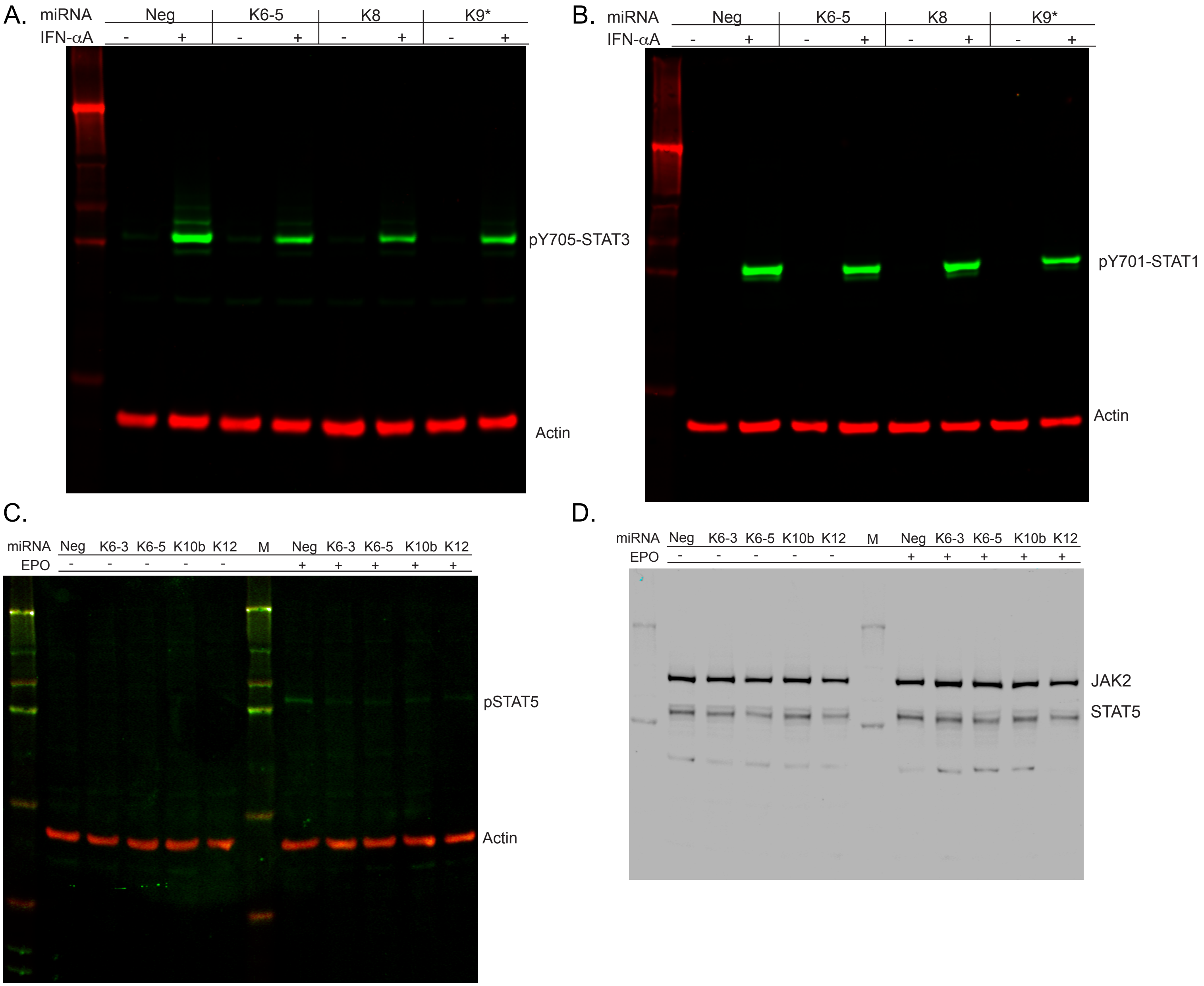

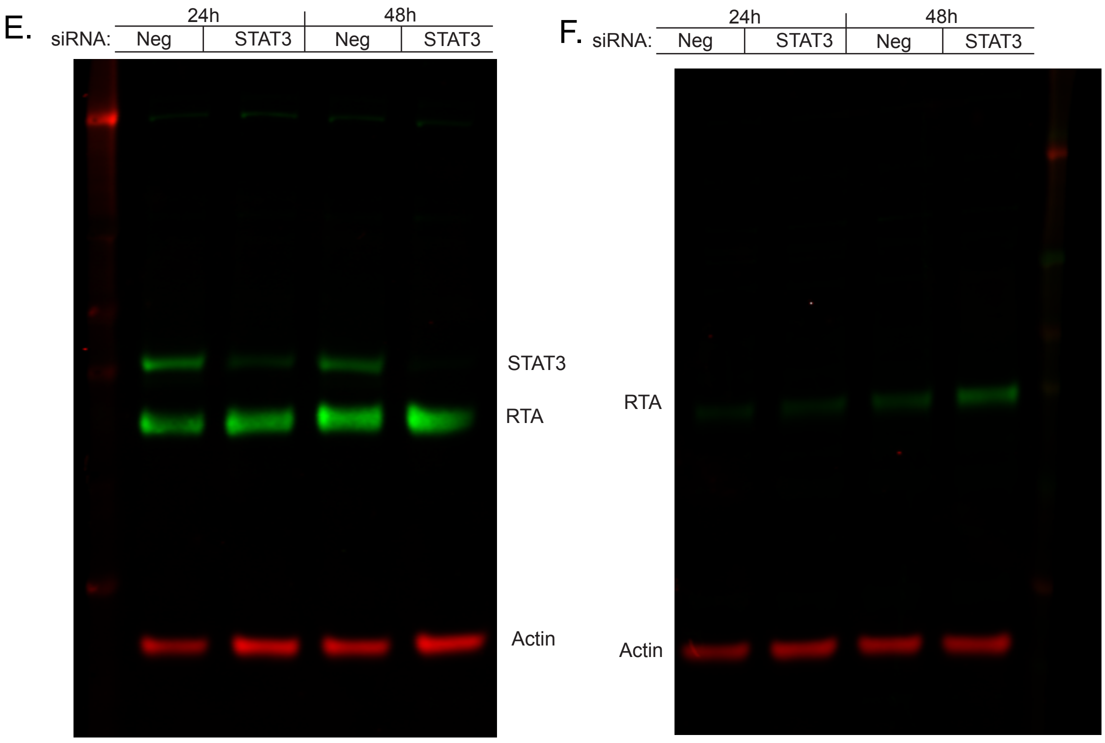

Supplementary Figure S3. Immunoblots in main figures.
A-B. Full blots for Figure 4.
C-D. Full blots for Figure 5.
E-F. Full blots for Figure 7.

Supplementary Table 1

Mean 48 hr.

3'UTR/miRNA	1	2	3	3star	4_3	4_5	5	6_3	6_5	7	8	9	9star	10a	10b	11	12-5
CTSZ				1.142										0.929			
IRAK1	0.548	0.944	0.738	0.621	1.056	0.894	0.841	0.691	0.953	0.817	0.865	0.576	0.829	1.025	0.932	0.920	
TNFRSF12A	1.123	1.251	1.188	0.956	1.337	1.244	1.142	0.913	1.130	1.114	1.253	1.166	1.112	0.480	1.077	1.078	
VCL								0.967	1.153		1.280						
EPOR		0.955	0.973									1.439	0.823		1.176		
TAGLN		1.165		1.009							1.168						
FOXK2					0.975						1.161						
JARID1B														0.882		0.395	
MET	0.974					1.756	1.514							0.683	1.481		
CORT												1.123	0.856	0.930			
DCBLD2										1.173	1.387	1.158					
CUL5										0.930	1.496			1.098	0.844		
ABI2	1.090	0.740	1.048	1.457										1.111	1.749		
PPM1E			0.638											0.620	0.916		
HMGB3							1.485		1.196					1.256	1.491		
PHF17										1.096				0.815			
LNX2	0.668		0.608											0.459			
OCRL			1.435	2.087										1.143	2.038		
TMEM97					1.102	1.193			0.660					0.757			
SENP7				1.214										0.775			
ESCO2	0.584	0.652						1.059		0.804							
CBX1		0.619					1.071		1.038					0.826	1.560		
ARID1A												0.851	0.813				
CDR2						1.365							1.148				
UPF3B				0.715					0.898					0.778			
CENPA				0.798				0.943				1.044			0.758		
CENPM	1.170							0.996		0.876	1.131			0.991			
GADD45B								1.204	1.031			0.785	1.001		1.351	0.755	
TPM1 EX13		1.254		1.169		1.141	0.664	1.099	0.998				0.848	0.886	0.820		
TPM1 EX14		1.730		1.041		1.379	1.058	1.023	1.091				0.999	0.859	0.877		
TPM1 EX15		0.866		0.877		1.279	1.016	0.533	0.854				0.783	0.587	0.484		
NISCH	1.086	0.835	0.395	0.882	0.443	1.161			0.425						1.315		
IL10RB										0.691		0.899	0.615				
PARP12		1.692										2.117	2.089	1.884	2.146		
DVL2			0.757	0.839	0.968	0.870	0.496				1.346		1.113	0.998	0.612		
COQ3								0.705		1.238		2.166		1.337	1.675		
INTS3					1.445					1.237						1.062	
PRKCD				1.341	1.427				0.646								
PDAP1								1.159	1.618		1.349				1.178		
UBA3									1.829				1.702	0.917	0.722		
VBP1					2.220								2.215	0.919			
N4BP2	0.760					1.177				0.642	0.732						
FANCC								1.021		0.823		0.869					
E2F2								0.817	1.079	1.329						0.445	
TNPO3		1.670			1.522	1.778		1.381							1.895		
UPF3B				0.885				0.654	0.905		0.975				1.359		
S100PBP		0.880				1.531								1.187	1.582		
NUP188											1.435	1.396	0.957				
SLC7A5	0.802										1.240	0.873					
NUSAP1										1.055				1.065	1.624		
RNF115					1.116		1.129				1.512				1.065		
BIRC5	0.792	1.323	0.917	1.400	1.344	1.116	0.998			0.907	0.907	0.789	0.793			0.995	0.697

Std.dev.

3'UTR/miRNA	1	2	3	3star	4_3	4_5	5	6_3	6_5	7	8	9	9star	10a	10b	11	12-5
CTSZ				0.224										0.117			
IRAK1	0.053	0.060	0.035	0.075	0.088	0.051	0.016	0.099	0.091	0.126	0.152	0.082	0.110	0.194	0.167	0.187	
TNFRSF12A	0.062	0.043	0.080	0.277	0.046	0.195	0.225	0.192	0.118	0.032	0.131	0.200	0.112	0.030	0.065	0.146	
VCL								0.047	0.007		0.199						
EPOR		0.033	0.021									0.111	0.096		0.069		
TAGLN		0.237		0.205							0.379						
FOXK2					0.302						0.309						
JARID1B														0.332		0.113	
MET	0.148					0.389	0.505							0.092	0.571		
CORT												0.316	0.095	0.137			
DCBLD2										0.528	0.543			0.414			
CUL5										0.082	0.637			0.116	0.172		
ABI2	0.238	0.167	0.170	0.397										0.286	0.425		
PPM1E			0.086											0.077	0.577		
HMGB3							0.134		0.330					0.241	0.046		
PHF17										0.167				0.018			
LNX2	0.067		0.036											0.133			
OCRL			0.059	0.412										0.219	0.516		
TMEM97					0.203	0.216			0.088					0.112			
SENP7				0.464										0.044			
ESCO2	0.068	0.057						0.253		0.133							
CBX1		0.118					0.383		0.338					0.258	1.068		
ARID1A												0.215	0.169				
CDR2						0.238							0.212				
UPF3B				0.247					0.200					0.134			
CENPA				0.060				0.204				0.377			0.210		
CENPM	0.288							0.081		0.146	0.092			0.228			
GADD45B								0.151	0.119			0.047	0.189		0.283	0.063	
TPM1 Ex13		0.180		0.062		0.212	0.048	0.069	0.165				0.166	0.093	0.140		
TPM1 Ex14		0.319		0.161		0.147	0.123	0.068	0.273				0.061	0.143	0.239		
TPM1 Ex15		0.172		0.155		0.227	0.178	0.032	0.155				0.121	0.084	0.084		
NISCH	0.046	0.107	0.046	0.066	0.052	0.076			0.087						0.133		
IL10RB										0.054		0.387	0.145				
PARP12		0.331										0.646	0.231	0.743	0.852		
DVL2			0.166	0.166	0.033	0.227	0.111				0.299		0.132	0.306	0.214		
COQ3								0.383		0.258		0.986		0.199	0.247		
INTS3					0.195					0.188					0.088		
PRKCD				0.330	0.122				0.090								
PDAP1								0.491	0.019		0.449				0.417		
UBA3									0.623				0.051	0.205	0.119		
VBP1					0.356								0.378	0.165			
N4BP2	0.115					0.107				0.119	0.070						
FANCC								0.150		0.182		0.123					
E2F2								0.130	0.126	0.048						0.082	
TNPO3		0.116			0.023	0.303		0.311							0.113		
UPF3B				0.270				0.065	0.155		0.305				0.284		
S100PBP		0.090				0.582								0.142	0.366		
NUP188											0.368	0.116	0.012				
SLC7A5	0.069										0.449	0.109					
NUSAP1										0.110				0.213	0.278		
RNF115					0.195		0.265				1.045				0.207		
BIRC5	0.030	0.067	0.092	0.394	0.051	0.062	0.196			0.372	0.074	0.066	0.070			0.135	0.098

**Oligo name**	**Oligo sequence (5' to 3')**
LNX2-a-f	AACTGATTTTCTGGTTTATTTCTACTGTAATCTCTCAATAA
LNX2-a-r	TATTGAGAGATTACAGTAGAAATAAACCAGAAAATCAGTTA
LNX2-b-f	AGATAAAACCATATTATTAAATACCTATATATCACAGCTAA
LNX2-b-r	TAGCTGTGATATATAGGTATTTAATAATATGGTTTTATCTA
LNX2-c-f	TGTCAGCAAAGCCAGTAACAACAGCGTGTACTGCCACTGTA
LNX2-c-r	ACAGTGGCAGTACACGCTGTTGTTACTGGCTTTGCTGACAA
UPF3B-b-f	GAATTTGGGTGGAGCAGAGTCGCTTTGAAGCCTTGTTCCG
UPF3B-b-r	CGGAACAAGGCTTCAAAGCGACTCTGCTCCACCCAAATTC
UBA3-a-f	AGTGCCATAGAGGCCAATATGCACAATATTAACTAATGCCA
UBA3-a-r	GGCATTAGTTAATATTGTGCATATTGGCCTCTATGGCACTA
NISCH-a-f	TTGCTGTTGCTGTTGGCATCTTGCTGCTAATCCTGAGGCTA
NISCH-a-r	AGCCTCAGGATTAGCAGCAAGATGCCAACAGCAACAGCAAA
SLC7A5-a-f	GTGTGGCTGGGACCTTCTTTATTCTGTGTTAATGGCTAACA
SLC7A5-a-r	GTTAGCCATTAACACAGAATAAAGAAGGTCCCAGCCACACA
LNX2-a-m1f	AACTGATTTTCTGGTTTATTTCTAGGCTAATCTCTCAATA
LNX2-a-m1r	TATTGAGAGATTAGCCTAGAAATAAACCAGAAAATCAGTT
LNX2-b-m1f	AGATAAAACCATATTATTAAATATACATATATCACAGCTA
LNX2-b-m1r	TAGCTGTGATATATGTATATTTAATAATATGGTTTTATCT
LNX2-c-m1f	TGTCAGCAAAGCCAGTAACAACAGCGTAGCCTGCCACTGT
LNX2-c-m1f	ACAGTGGCAGGCTACGCTGTTGTTACTGGCTTTGCTGACA
NISCH-a-m1f	TTGCTGTTGCTGTTGGCATCTTGCATGTAATCCTGAGGCT
NISCH-a-m1r	AGCCTCAGGATTACATGCAAGATGCCAACAGCAACAGCAA

Supplementary Table 3

Interactions Report

#	From: Network Object "FROM"	From: Object Type	To: Network Object "TO"	To: Object Type	Effect	Mechanism	Homo sapiens	Link Info	Pubmed IDs: References
1	STAT3	Transcription factor	GADD45 beta	Generic binding protein	Activation	Transcription regulation	x	putitatuve Stat3 binding site on GADD45 beta was validated by measuring in vitro binding and in vivo occupancy.	19282476;20467778
2	BIRC5	Generic binding protein	STAT3	Transcription factor	Inhibition	Binding	x	Acetylated BIRC5 binds to the N-terminal transcriptional activation domain of the STAT3 dimer and represses STAT3 transactivation of target gene promoters.	20826784
3	STAT3	Transcription factor	N4BP2	Generic kinase	Unspecified	Transcription regulation	x	Stat3 binds to gene N4BP2 promoter.	18065416
4	PKC-delta	Protein kinase	HGF receptor (Met)	Receptor with enzyme activity	Inhibition	Phosphorylation	x	PKC-delta phosphorylates HGF receptor (Met) and decreases its activity.	15075332
5	IRAK1	Protein kinase	STAT3	Transcription factor	Activation	Phosphorylation	x	IRAK1 phosphorylates STAT3 and increases its activity.	15465816
6	STAT3	Transcription factor	BIRC5	Generic binding protein	Activation	Transcription regulation	x	Activated Stat3 signaling contributes to breast cancer progression and resistance to chemotherapy by, at least in part, inducing expression of the antiapoptotic protein, BIRC5.	13678425;16397018;16529541;17533050;17934481;18075512;18242580;19744341;19880237;19937797;21521803
7	STAT3	Transcription factor	STAT3	Transcription factor	Activation	Binding	x	Dimeric and tetrameric STAT3 complexes formation is required for transcriptional activity of STAT3.	7701321;8608222;8782827;10458605;11438543;18849387;19053881
8	HGF receptor (Met)	Receptor with enzyme activity	GRB2	Generic binding protein	Activation	Binding	x	HGF receptor (Met) directly binds to GRB2 and triggers RAS pathway.	7511210;8662889;9184210;9660480;10069803;11430831;11439336;11900489;12766170;15546961;16724056;17065554;17449471;18003605;21784853
9	Epo receptor	Generic receptor	Epo receptor	Generic receptor	Activation	Binding	x	Our results indicate that Epo exerts its antiapoptotic effects on differentiated SH-SY5Y and PC-12 cells through the standard stoichiometry of one molecule of Epo binding to two EpoR subunits, comprising the	9793257;10912517;11287756;17045782
10	HGF receptor (Met)	Receptor with enzyme activity	STAT3	Transcription factor	Activation	Binding	x	HGF stimulates recruitment of Stat-3 to the receptor, tyrosine phosphorylation, nuclear translocation and binding to the specific promoter element SIE.	9440692;10069803;15254691;15485908;16724056
11	PKC-delta	Protein kinase	STAT3	Transcription factor	Activation	Phosphorylation	x	Stat3 tyrosine phosphorylation is required for Stat3 association with PKC-delta.	9343414;9872331;10446219;10521505;10770494;11335711;12361954;12576423;12763138;14551213;15380937;16418226;16951338;17283176
12	E2F2	Transcription factor	BIRC5	Generic binding protein	Unspecified	Transcription regulation	x		15271987;17916908
13	HGF receptor (Met)	Receptor with enzyme activity	HGF receptor (Met)	Receptor with enzyme activity	Activation	Phosphorylation	x	HGF receptor (Met) phosphorylates HGF receptor (Met) and increases its activity.	11779195
14	Epo receptor	Generic receptor	STAT3	Transcription factor	Activation	Binding	x	Epo receptor interacts with STAT3 and activates it	11756159;15752978

BIRC5 3'UTR data	24h	SD	N	48h	SD	N
Neg1	1.00	0.00	3	1.00	0.00	3
miR-K1	0.99	0.06	3	0.79	0.03	3
miR-K2	0.97	0.25	3	1.32	0.07	3
miR-K3	0.87	0.12	3	0.92	0.09	3
miR-K3*	1.13	0.35	3	1.40	0.39	3
miR-K4-3	0.98	0.23	3	1.34	0.05	3
miR-K8	0.98	0.17	3	0.91	0.07	3
mir-K9	0.94	0.17	3	0.79	0.07	3
miR-K11	0.80	0.21	3	0.99	0.13	3
miR-K12-5	0.63	0.16	3	0.70	0.10	3

miRNAs that didn’t show repression

Neg1	1.00	0.00	3	1.00	0.00	3
miR-K6-3	1.19	0.04	3	1.17	0.28	3
miR-K6-5	1.69	0.13	3	2.54	1.07	3
miR-K10b	1.46	0.05	3	1.61	0.53	3
miR-K12	1.44	0.11	3	1.90	0.67	3

Red if $p<0.05$
